# Analyzing the mahakam river water quality using the geographically weighted panel regression model

**DOI:** 10.1016/j.mex.2025.103773

**Published:** 2025-12-19

**Authors:** Zabrina Nathania Fauziyah, Suyitno Suyitno, Darnah, Memi Nor Hayati, Meirinda Fauziyah

**Affiliations:** Statistics Study Program, Department of Mathematics, Faculty of Mathematics and Natural Sciences, Mulawarman University, Indonesia

**Keywords:** Akaike information criterion, Biochemical oxygen demand, Demean data, Fixed effect model, Spatial heterogeneity panel data

## Abstract

This study discusses the geographically weighted panel regression (GWPR) model. GWPR is an extension of geographically weighted regression model, designed for spatially heterogeneous panel data. In this study, GWPR model is applied to panel data on biochemical oxygen demand (BOD) in Mahakam River water 2022–2024. The model is estimated at each spatial location using a fixed effects model (FEM) as the global model, with temporal effects addressed through a demeaning transformation. All statistical analyses and spatial processing are conducted using R software, GNU Octave, QGIS, and Google Earth. This study aims to map factors influencing Mahakam River water BOD using GWPR model. The results indicate that GWPR outperforms FEM, with AIC = -60.6419, $R^{2}=80.321\%$, and root mean square error of 0.7122. The factors influencing BOD include temperature, water pH, color degree, nitrate, ammonia, total suspended solids, and sulfate.

• *We present a GWPR model using FEM as global model, applied to the spatially heterogeneous panel data, namely demeaned Mahakam River water BOD data 2022–2024.*
• *The mapping of factors influencing BOD is analyzed locally using GWPR model.*
• *The optimal adaptive bandwidth is determined using Akaike Information Criterion, and model goodness-of-fit is evaluated using the coefficient of determination and root mean square error.*

## Graphical abstract

**Figure:**
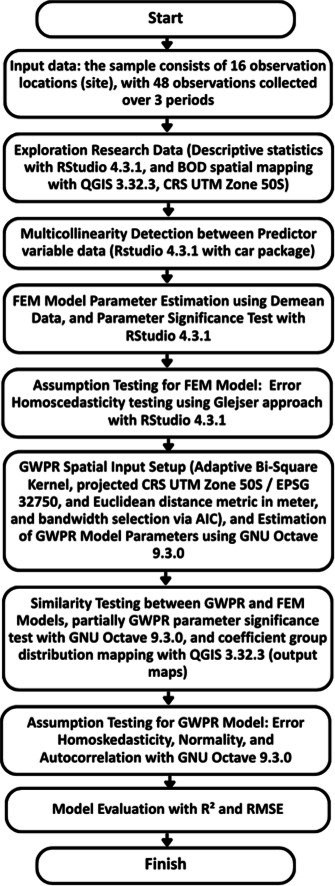

**Specifications table**

**Table:** 

**Subject area**	Mathematics and Statistics
**More specific subject area**	*Geographically Weighted Regression Model; Environmental Health*
**Name of your method**	*Geographically Weighted Panel Regression Model and Its Application*
**Name and reference of original method**	*None*
**Resource availability**	*The research data is secondary data, namely the Analyze Report of the Surface Water Data Quality Monitoring from the Life Environmental Department of the East Kalimantan Province 2022–2024. The research sample size was 16 observation points with 48 observation data within 3 periods in 2022–2024. The research data consists of the response variable data, namely BOD*(y)*, and predictor variable data, namely temperature*$\left(x_{1}\right)$*, water* pH$\;(x_{2})$*, water color degree*$\left(x_{3}\right)$*, nitrate concentration*$\left(x_{4}\right)$*, ammonia concentration*$\left(x_{5}\right)$*, total suspended solid or TSS*$\left(x_{6}\right)$*, sulfate concentration*$\left(x_{7}\right)$*, also coordinate data of observation location points. Data processing and analysis were performed using RStudio 4.3.1, GNU Octave 9.3.0, and QGIS 3.32.3.*

## Background

Generally, the data used in the linear regression modeling is the cross-sectional data [1]. However, many data in the field of environmental health are found in the form of panel data, namely the combination of cross-sectional and time series data, and they are spatial heterogeneity data [[2], [3], [4]] Therefore, it is necessary to develop the geographically weighted regression (GWR) model to a geographically weighted panel regression (GWPR) model. The GWPR model in this research is applied to spatial heterogeneity panel data, namely biochemical oxygen demand (BOD) of the Mahakam River water in 2022–2024. BOD is the amount of oxygen consumed by microorganisms to decompose organic matter in water. BOD is one of the indicators of river water quality, in which the high BOD level indicates the declining the water quality [5]. The raw source of drinking water for the majority of East Kalimantan people is Mahakam River water. Therefore, the quality of Mahakam River water must be maintained. However, there are many activities along the Mahakam River basin, such as water transportation line, mining, industry, restaurant, hotel, and residential area, which have the potential to increase the river water BOD [6]. The increasing river water BOD can degrade the river water quality, therefore the Mahakam River water BOD level needs to be maintained and monitored [7].

One effort to maintain the Mahakam River BOD statistically is proposed in the research, namely predicting the BOD locally and analyzing the factors influencing it through the GWPR model. The proposed global model of the GWPR model in this research is FEM. Parameter estimation method proposed in this research is the weighted least square (WLS), with spatial weight is calculated using the adaptive bi-square function based on demean data. The optimum bandwidth selection in this research uses the Akaike information criterion (AIC) in which the selection of the optimum bandwidth of GWR model in the previous researches generally using cross validation (CV) [[8], [9], [10], [11]] or generalized cross validation (GCV) approach [10,12,13] Based on FEM, the parameter estimation of the GWPR model in this research is conducted at every observation location point ignoring the time period. The measure of the model goodness in this research is the coefficient of determination (R^2^), and the root mean square error (RMSE), including AIC. The analysis of the water BOD has been conducted in previous researches with different methods such as, machine learning and topological kriging [14] artificial neural network [15] support vector regression [16] and Graphical analysis and K-nearest neighbor analysis [17].

## Method details

*1. Linear Regression Model*

Linear regression analysis is a statistical method to model the linear relationship between a response variables and one or more predictor variables, to measure the influence of the predictor variable to the response variable, and to predict the value of response variable [18]. Parameters estimation of the linear regression model carried out using the ordinary least square (OLS) method by minimizing the sum of squared errors [1]. The general form of the multiple linear regression model stating the relationship a response variable and p predictor variables can be expressed as

(1) $$y_{i}=b_{0}+b_{1}x_{i1}+b_{2}x_{i2}+\ldots +b_{p}x_{ip}+\varepsilon_{i},i=1,2,\ldots ,\;n$$

with $y_{i}$ is the response variable data at the i-th observation point, $x_{ik}$​ is the data of k-th predictor at i-th observation point, $b_{0}$ is intersept, $b_{k};k=1,2,\ldots p$ is the regression parameter, and $\varepsilon_{i}$ is the error term [1]. The regression model given by Eq. (1) can be expressed in matrix form as

(2) y=Xb+ɛ

*with*

(3) $$y=\left[\begin{array}{c} y_{1}\\ y_{2}\\ \vdots \\ y_{n} \end{array}\right],\;X=\left[\begin{array}{ccccc} 1 & x_{11} & x_{12} & \cdots & x_{1p}\\ 1 & x_{21} & x_{22} & \cdots & x_{2p}\\ \vdots & \vdots & \vdots & \ddots & \vdots \\ 1 & x_{n1} & x_{n2} & \cdots & x_{np} \end{array}\right],b=\left[\begin{array}{c} b_{0}\\ b_{1}\\ \vdots \\ b_{p} \end{array}\right],and\;ɛ=\left[\begin{array}{c} \varepsilon_{1}\\ \varepsilon_{2}\\ \vdots \\ \varepsilon_{n} \end{array}\right].$$

Using the OLS method, the estimator of the regression parameter is given by

(4) $$\hat{b}={\left(X^{T}X\right)}^{-1}X^{T}y.$$

The classical linear regression model is based on several assumptions, including the relationship between predictors and the response is linear, the error has to follow the normal distribution with zero mean and constant variance (homoscedasticity), and the Inter-errors are independent (non-autocorrelation) [19].

*2. Significance Test of Parameter of Linear Regression*

The significance test of parameter of linear regression model consist of the simultaneous testing and partial testing. The simultaneous testing useful to analyze the model goodness of fit, with the hypothesis is

$H_{0}$: $b_{1}=b_{2}=\ldots =b_{p}=0$ (the regression model if not fit)

$H_{1}$: there is at least one $b_{k}\neq 0;k=1,2,\ldots ,\;p$ (the regression model is fit)

The test statistic is given by

(5) $$F_{1}=\frac{\text{MSR}}{\text{MSE}},$$

with

(6) $$\text{MSR}=\frac{\sum_{i=1}^{n}{\left({\hat{y}}_{i}-\bar{y}\right)}^{2}}{p},$$

(7) $$\text{MSE}=\frac{\sum_{i=1}^{n}{\left(y_{i}-{\hat{y}}_{i}\right)}^{2}}{n-p-1}.$$

The test statistic $F_{1}$ given by Eq. (5) follows F distribution, namely $F_{1}∼F_{\left(p;n-p-1\right)}$. The critical region of the simultaneous testing is to reject $H_{0}$ at the significance level ofα if only if $F_{1}>F_{\left(1-\alpha ;p;n-p-1\right)}$, or to reject $H_{0}$ if only if $p_{1}<\alpha$ [1] with

(8) $$p_{1}=P\left(F>F_{1}\right)=1-F\left(F_{1}\right)$$

The second parameter testing is partial testing. Partial testing useful to analyze the effect every predictor variable to the response variable. The hypothesis for testing parameter $b_{k}$ with certain k (k=1,2,…,p) is

$H_{0}$: $b_{k}=0$

$H_{1}$: $b_{k}\neq 0$

The test statistic is given by

(9) $$T_{1}=\frac{{\hat{b}}_{k}}{\sqrt{\text{var}\left({\hat{b}}_{k}\right)}},$$

with $\text{var}({\hat{b}}_{k})\;$is the k-th element of the major diagonal of variance-covariance matrix of $\hat{b}$ [1] given by

(10) $$\text{var}\left(\hat{b}\right)={\left(X^{T}X\right)}^{-1}.$$

The test statistic $T_{1}$ given by Eq. (9) follows t distribution, namely $T_{1}∼t_{\left(n-p-1\right)}$. The critical region for partial testing is to reject $H_{0}$ at the significance level ofα if only if $|T_{2}|>t_{1-\frac{\alpha }{2};\left(n-p-1\right)}$, or to reject $H_{0}$ if only if $p_{2}<\alpha$ [1] with

(11) $$p_{2}=2P\left(T_{v}>T_{1}\right)=2\left(1-F\left(T_{1}\right)\right).$$

*3. Panel Regression Model*

Panel regression model is a regression model used to analyze panel data. Panel data is a combination of cross-sectional and time series data obtained through repeated observations of the same unit during a certain period [20]. There are three general approaches in panel regression, namely the Common Effect Model (CEM), Random Effect *Model (REM), and Fixed Effect Model (FEM)* [[21], [22], [23]] *This research focuses on the FEM.*

The FEM assumes that each unit has the unique characteristics. These characteristics are constant for model parameters over time, and the intercept are different for each observation location points [21]. Parameter estimation of FEM can be conducted in several approaches such as least squares dummy variable (LSDV) [24] ordinary least squares (OLS) using demean data [25] and OLS for two-way fixed effects [26]. Parameter estimation of FEM in this research uses the OLS method with demean data, namely adopting parameter estimation method of linear regression using the demean data [27]. The general model of FEM is

(12) $$y_{it}^{*}=\beta_{0_{i}}+\beta_{1}x_{it1}+\beta_{2}x_{it2}+\ldots +\beta_{p}x_{itp}+\varepsilon_{it}^{*};\;i=1,2,\ldots ,\;n;\;t=1,2,\ldots ,\;\tau .$$

The first step to construct the FEM model with within estimator is to formulate the average model, that is

(13) $${\bar{y}}_{i}=\beta_{0_{i}}+\beta_{1}{\bar{x}}_{i1}+\beta_{2}{\bar{x}}_{i2}+\ldots +\beta_{p}{\bar{x}}_{ip}+{\bar{\varepsilon }}_{it},\;i=1,2,\ldots ,\;n$$

with

(14) $${\bar{y}}_{i}=\frac{1}{\tau }\sum_{t=1}^{\tau }y_{it},\;{\bar{x}}_{ik}=\frac{1}{\tau }\sum_{t=1}^{\tau }x_{itk},\;and\;{\bar{\varepsilon }}_{i}=\frac{1}{\tau }\sum_{t=1}^{\tau }\varepsilon_{it},k=1,2,\ldots ,\;p.$$

Subtracting Eq. (12) by Eq. (13) is obtained the FEM with within estimator model which has an expression

(15) $$y_{it}^{*}=\beta_{1}x_{it1}^{*}+\beta_{2}x_{it2}^{*}+\ldots +\beta_{p}x_{itp}^{*}+\varepsilon_{it}^{*},\;t=1,2,\ldots ,\;\tau$$

with

(16) $$y_{it}^{*}=\left(y_{it}-{\bar{y}}_{i}\right),\;x_{itk}^{*}=\left(x_{itk}-{\bar{x}}_{ik}\right),\;and\;\varepsilon_{it}^{*}=\left(\varepsilon_{it}-{\bar{\varepsilon }}_{i}\right).$$

The data of $y_{it}^{*}$, $x_{itk}^{*}$, and $\varepsilon_{it}^{*}$ resulting by a data transformation given by Eq. (16) is called demeaned data [27]. Referring Eq. (15), the FEM can be expressed in the matrix form as

(17) $$y^{*}=X^{*}\beta +{ɛ}^{*},$$

with(18)Based on demean data given by Eq. (16), the parameter estimation method of FEM is OLS, and it is obtained

(19) $$\begin{array}{c} \hat{\beta }={\left({X^{*}}^{T}X^{*}\right)}^{-1}{X^{*}}^{T}y^{*} \end{array}$$

*4. Significance Test of FEM Parameter*

The significance test of the FEM parameter consisted of the simultaneous testing, and the partial testing. The simultaneous testing is useful to analyze the model goodness of fit, with the hypothesis is

$H_{0}$: $\beta_{1}=\beta_{2}=\ldots =\beta_{p}=0$ (the FEM is not fit)

$H_{1}$: there is at least one $\beta_{k}\neq 0;k=1,2,\ldots ,\;p$ (the FEM is fit)

The test statistic is given by

(20) $$F_{2}=\frac{\text{MSR}}{\text{MSE}},$$

*with*

(21) $$\text{MSR}=\frac{\sum_{i=1}^{n}\sum_{t=1}^{\tau }{\left({\hat{y}}_{it}^{*}-{\bar{y}}^{*}\right)}^{2}}{p},$$

(22) $$\text{MSE}=\frac{\sum_{i=1}^{n}\sum_{t=1}^{\tau }{\left(y_{it}^{*}-{\hat{y}}_{it}^{*}\right)}^{2}}{n\tau -n-p}.$$

The test statistic $F_{2}$ given by Eq. (20) follows F distribution, namely $F_{2}∼F_{\left(p;n\tau -n-p\right)}$. The critical region for simultaneous testing is to reject $H_{0}$ at the significance levelα if only if $F_{2}>F_{\left(1-\alpha ;p;n\tau -n-p\right)}$, or to reject $H_{0}$ if only if $p_{3}<\alpha$ [20] with

(23) $$p_{3}=P\left(F_{v}>F_{2}\right)=1-F\left(F_{2}\right)$$

The partial testing is useful to analyze the effect every predictor variable to the response variable. The hypothesis for testing parameter $\beta_{k}$ for certain k (k=1,2,…,p) is

$H_{0}$: $\beta_{k}=0$

$H_{1}$: $\beta_{k}\neq 0$

The test statistic is given by

(24) $$T_{2}=\frac{{\hat{\beta }}_{k}}{\sqrt{\text{var}\left({\hat{\beta }}_{k}\right)}},$$

with $\text{var}({\hat{\beta }}_{k})$ is the k-th element of the major diagonal of variance-covariance matrix of $\hat{\beta }$ given by

(25) $$\begin{array}{c} \text{var}\left(\hat{\beta }\right)={\left({X^{*}}^{T}X^{*}\right)}^{-1}. \end{array}$$

The test statistic $T_{2}$ given by Eq. (24) follows t distribution, namely $T_{2}∼t_{\left(n\tau -p\right)}$. The critical region for partial testing is to reject $H_{0}$ at the significance levelα if only if $|T_{2}|>t_{1-\frac{\alpha }{2};\left(n\tau -p\right)}$, or to reject $H_{0}$ if only if $p_{4}<\alpha$ [20] with

(26) $$p_{4}=2P\left(T_{v}>T_{2}\right)=2\left(1-F\left(T_{2}\right)\right)$$

*5. The Assumption Testing of the FEM Model*

In this study, the proposed global model is limited to the FEM, therefore, the assumption tests presented in this section are limited to homoscedasticity, non-autocorrelation, and normality of the error term. Furthermore, the Hausman test for identifying the best global model was not conducted, and non-stationarity tests were also not performed. Assumption testing of the FEM model is necessary to ensure that the basic assumptions of the FEM model are satisfied and to obtain precise analysis. One key assumption of FEM model is that the error term must follow a normal distribution with zero mean and constant variance, i.e., homoscedasticity. Homoscedasticity implies that the variance of the error term is constant, namely, $var\left(\varepsilon_{it}\right)=\sigma^{2}$ fori=1,2,…,n, and t=1,2,…,τ [28]. One method to test homoscedasticity is the Glejser test, under the following hypotheses:

$H_{0}$: $\sigma_{1}^{2}=\sigma_{2}^{2}=\ldots =\sigma_{n}^{2}=\sigma^{2}$ (the variance of the error term is constant)

$H_{1}$: there is at least one$\;\sigma_{i}^{2}\neq \;\sigma_{j}^{2},\;i\neq j$, i,j=1,2,…,n (the variance of the error term is not constant)

Homoscedasticity testing of error term is also applied to data with spatial heterogeneity.

The test statistic is given by:

(27) $$F_{3}=\frac{\left({\hat{\varphi }}^{T}X^{*T}\delta -n{\bar{e}}^{2}\right)/p}{\left(\delta^{T}\delta -{\hat{\varphi }}^{T}X^{*T}\delta \right)/\left(n\tau -n-p\right)},$$

where $\hat{\varphi }$ is the OLS estimator of the auxiliary regression model, and $\delta ={\left[\begin{array}{ccc} |\varepsilon_{i}^{*}| & |\varepsilon_{2}^{*}| & \begin{array}{cc} \ldots & |\varepsilon_{n}^{*}| \end{array} \end{array}\right]}^{T},$ with $\;\varepsilon_{i}^{*}$ is defined in Eq. (18). The test statistic $F_{3}$ given by Eq. (27) follows F distribution, specifically $F_{3}∼F_{\left(p;n\tau -n-p\right)}$. The critical region for homoscedasticity test is defined such that $H_{0}$ is rejected at the significance levelα if and only if $F_{3}>F_{\left(1-\alpha ;p;n\tau -n-p\right)}$, or equivalently, if and only if $p_{5}<\alpha$ [11] with

(28) $$p_{5}=P\left(F_{v}>F_{3}\right)=1-F\left(F_{3}\right)$$

One method that can be used to test the data normality is the Jarque-Bera (JB) test, which evaluates normality based on skewness(S) and kurtosis (K) [29]. The hypothesis of the normality testing is

$H_{0}$: Errors $\left(\varepsilon_{it}^{*}\right)$ follows a normally distribution with $E(\varepsilon_{it}^{*})=0$, i=1,2,…,n,andt=1,2,…,τ.

$H_{1}$: Error $\left(\varepsilon_{it}^{*}\right)$ doesn’t follow normal distribution

The test statistic is given by

(29) $$JB=n\left[\frac{S^{2}}{6}+\frac{{\left(K-3\right)}^{2}}{24}\right],$$

with

(30) $$S=\frac{\frac{1}{n}\sum_{i=1}^{n}\sum_{t=1}^{\tau }{\left(\varepsilon_{it}^{*}\right)}^{3}}{{\left(\frac{1}{n}\sum_{i=1}^{n}\sum_{t=1}^{\tau }{\left(\varepsilon_{it}^{*}\right)}^{2}\right)}^{3/2}},$$

(31) $$K=\frac{\frac{1}{n}\sum_{i=1}^{n}\sum_{t=1}^{\tau }{\left(\varepsilon_{it}^{*}\right)}^{4}}{{\left(\frac{1}{n}\sum_{i=1}^{n}\sum_{t=1}^{\tau }{\left(\varepsilon_{it}^{*}\right)}^{2}\right)}^{2}},$$

The test statistic JB given by Eq. (29) follows $\chi^{2}$ distribution, namely $JB∼\chi_{2}^{2}$. The critical region for normality testing is to reject $H_{0}$ at the significance level ofα if only if $JB>\chi_{\left(1-\alpha ;\;2\right)}^{2}$, or to reject $H_{0}$ if only if $p_{4}<\alpha$,with

(32) $$p_{6}=P\left(X_{v}>JB\right)=1-F\left(JB\right),$$

Error autocorrelation testing of the FEM is necessary to ensure that the model errors are spatially independent. The presence of spatial autocorrelation indicates that Error at nearby locations are correlated, which violates the independence assumption and implies that spatial effects are not fully captured by the model. One method that can be used to detect spatial autocorrelation is the Moran’s I test [30]. The hypothesis of the Moran’s I test is

H₀ : the FEM Error are spatially independent

H₁ : the FEM Error are spatially dependent

The test statistic of Moran’s I is given by

(33) $$I=\frac{n}{S_{0}}\frac{\sum_{i}\sum_{j}w_{ij}\left(e_{i}^{*}-{\bar{e}}^{*}\right)\left(e_{j}^{*}-{\bar{e}}^{*}\right)}{\sum_{i}{\left(e_{i}^{*}-{\bar{e}}^{*}\right)}^{2}}$$

with $e_{i}^{*}$ and $e_{j}^{*}$ are the FEM Error at locations i and j, respectively ${\bar{e}}^{*}$is the mean of the Error, $w_{ij}$ is the spatial weight element between locations i and j is the number of locations, and $S_{0}=\sum_{i}\sum_{j}w_{ij}$ [30].

The expected value of Moran’s I under the null hypothesis is E[I]=−1/(n−1). If the calculated Moran’s I is significantly different fromI, the null hypothesis is rejected, indicating the presence of spatial autocorrelation. The decision rule for the Moran’s I test is to reject H_0_ at significance level α if $|Z_{I}|>Z_{\left(1-\alpha /2\right)}$, with

(34) $$Z_{I}=\frac{I-E\left[I\right]}{\sqrt{Var\left(I\right)}}$$

where Var(I) is the variance of the Moran’s I statistic [30].

*6. Multicollinearity Detection Between Predictor Variables*

Multicollinearity is a condition in which there is a linear relationship between predictor variables in the regression model. The method that can be used to identify multicollinearity is the variance inflation factor (VIF). VIF value >10 indicates that there is a multicollinearity problem between the predictor variables in the regression model [31]. The VIF value can be calculated through the following equation

(35) $$VIF_{k}=\frac{1}{1-R_{k}^{2}},$$

with $VIF_{k}$ is the VIF value of the k-th predictor variable, and $R_{k}^{2}$ is the coefficient of determination of the auxiliary regression. The auxiliary regression is the regression model of $x_{k}^{*}\;$regressed to the other predictor variables [11].

*7. Spatial Weighting Function*

Spatial weight in parameter estimation is a representation of spatial effect to model. Spatial calculated using a weighting function, one of spatial weighting function is the adaptive bi-square function [32] defined by

(36) $$w_{ij}=\left\{ \begin{array}{c} {\left(1-{\left(\frac{d_{ij}}{h_{i}}\right)}^{2}\right)}^{2},if\;d_{ij}\leq h\\ 0,\;otherwise \end{array}\right.$$

with $w_{ij}$ is spatial weight given by the observation data at j-th observation location point for parameter estimation of GWPR model at i-th observation point, $h_{i}$ represents the adaptive bandwidth at i-th observation location point, and $d_{ij}$ is the Euclidean distance between i th observation point and j-th observation point, which given by

(37) $${\left(d_{ij}\right)}^{2}={\left(u_{i}-u_{j}\right)}^{2}+{\left(v_{i}-v_{j}\right)}^{2}$$

The optimum bandwidth ($h_{i}$) selection in Eq. (36) is a crucial problem. The bandwidth that is too large will result in spatial weights equal to 1 making the model is equivalent to the global model. Conversely, the bandwidth that is too small result in the spatial weights will be close to zero resulting the bias model. The optimal bandwidth in this research is found using the akaike information criterion (AIC), with its value is calculated based on the formula

(38) $$\text{AIC}=n\tau \log\left(\frac{\sum_{i=1}^{n}\sum_{t=1}^{\tau }{\left(y_{it}^{*}-{\hat{y}}_{it}^{*}\right)}^{2}}{n\tau }\right)+2p,$$

with n is the sample size, τ is period number, and p is the number of the parameter in the model. The optimum bandwidth is a bandwidth resulting a model with the minimum AIC value [33]. The measure of the model goodness in the research is the coefficient of determination (R²), and the Root Mean Square Error (RMSE) [34].

*8. Geographically Weighted Panel Regression Model*

Geographically weighted panel regression (GWPR) is a geographically weighted regression (GWR) model applied to heterogeneity spatial panel data. Based on GWR model, the GWPR model parameter is estimated at every observation location point. The global model in this research is fixed effect model (FEM). Suppose, the coordinate of every observation point is known, and it is stated in the row vector $\left(u_{i},v_{i}\right)$, with $u_{i}$ is latitude and $v_{i}$ is longitude of the i th observation location point [21]. The general form of the GWPR model at the i-th observation location point at the t-th time is

(39) $$y_{it}^{*}=\beta_{1}\left(u_{i},v_{i}\right)x_{it1}^{*}+\beta_{2}\left(u_{i},v_{i}\right)x_{it2}^{*}+\ldots +\beta_{p}\left(u_{i},v_{i}\right)x_{itp}^{*}+\varepsilon_{it}^{*},\;i=1,2,\ldots ,n;\;t=1,2,\ldots ,\;\tau .$$

In matrix form, the model given by Eq. (37) can be written as

(40) $$y^{*}=X^{*}\beta \left(u_{i},v_{i}\right)+{ɛ}^{*}.$$

Parameter estimation method of the GWPR model is the weighted least square (WLS), with the parameter estimator is given by

(41) $$\hat{\beta }\left(u_{i}.v_{i}\right)={\left({X^{*}}^{T}W\left(u_{i},v_{i}\right)X^{*}\right)}^{-1}{X^{*}}^{T}W\left(u_{i},v_{i}\right)y^{*};i=1,2,\ldots ,n$$

and$\;W(u_{i},v_{i})$ is the spatial weighting matrix for parameter estimation of GWPR model ati-th observation location point, and it is given by

(42) $$W\left(u_{i}.v_{i}\right)=diag\left[\begin{array}{ccc} w_{i11} & w_{i21} & \begin{array}{cc} \ldots & \begin{array}{ccc} w_{in1} & w_{i12} & \begin{array}{ccc} w_{i22} & \ldots & \begin{array}{ccc} w_{in2} & \ldots & \begin{array}{ccc} w_{i1\tau } & w_{i2\tau } & \begin{array}{cc} \ldots & w_{in\tau } \end{array} \end{array} \end{array} \end{array} \end{array} \end{array} \end{array}\right].$$

*9. Similarity Testing between GWPR and FEM Models*

The Similarity testing is to test whether the GWPR model and FEM model are identic, with the hypothesis is

$H_{0}$: $\beta_{k}\left(u_{i}.v_{i}\right)=\beta_{k},\;i=1,\;2,\;\ldots ,\;n;\;k=1,2,\ldots ,\;p$ (The FEM and GWPR models are identic)

$H_{1}$: there is at least one $\beta_{k}\left(u_{i}.v_{i}\right)\neq \beta_{k},\;i=1,\;2,\;\ldots ,\;n;k=1,\;2,\ldots ,\;p$ (The FEM and GWPR models are not identic)

The test statistic is given by

(43) $$F_{4}=\frac{SSE_{G}\left(H_{0}\right)/df_{1}}{SSE_{G}\left(H_{1}\right)/df_{2}},$$

*with*

(44) $$SSE_{G}\left(H_{0}\right)={\left(y^{*}-{\hat{y}}^{*}\right)}^{T}\left(y^{*}-{\hat{y}}^{*}\right)=y^{{*}^{T}}\left(I^{*}-H^{*}\right)y^{*},$$

(45) $$SSE_{G}\left(H_{1}\right)=y^{{*}^{T}}{\left(I^{*}-L^{*}\right)}^{T}\left(I^{*}-L^{*}\right)y^{*}.$$

where $H^{*}$ given by the projection matrices for FEM model defined by

(46) $$H^{*}=X^{*}{\left({X^{*}}^{T}X^{*}\right)}^{-1}{X^{*}}^{T}$$

$L^{*}$ given by the projection matrices for GWPR model defined by

(47) $$L^{*}=\left[\begin{array}{c} x_{11}^{*T}{\left(X^{T}W\left(u_{1}.v_{1}\right)X\right)}^{-1}X^{T}W\left(u_{1}.v_{1}\right)\\ x_{21}^{*T}{\left(X^{T}W\left(u_{2}.v_{2}\right)X\right)}^{-1}X^{T}W\left(u_{2}.v_{2}\right)\\ \begin{array}{c} \vdots \\ x_{n1}^{*T}{\left(X^{T}W\left(u_{n}.v_{n}\right)X\right)}^{-1}X^{T}W\left(u_{n}.v_{n}\right)\\ \begin{array}{c} x_{12}^{*T}{\left(X^{T}W\left(u_{1}.v_{1}\right)X\right)}^{-1}X^{T}W\left(u_{1}.v_{1}\right)\\ x_{22}^{*T}{\left(X^{T}W\left(u_{2}.v_{2}\right)X\right)}^{-1}X^{T}W\left(u_{2}.v_{2}\right)\\ \begin{array}{c} \vdots \\ x_{n2}^{*T}{\left(X^{T}W\left(u_{n}.v_{n}\right)X\right)}^{-1}X^{T}W\left(u_{n}.v_{n}\right)\\ \begin{array}{c} \vdots \\ x_{1\tau }^{*T}{\left(X^{T}W\left(u_{1}.v_{1}\right)X\right)}^{-1}X^{T}W\left(u_{1}.v_{1}\right)\\ \begin{array}{c} x_{2\tau }^{*T}{\left(X^{T}W\left(u_{2}.v_{2}\right)X\right)}^{-1}X^{T}W\left(u_{2}.v_{2}\right)\\ \vdots \\ x_{n\tau }^{*T}{\left(X^{T}W\left(u_{n}.v_{n}\right)X\right)}^{-1}X^{T}W\left(u_{n}.v_{n}\right) \end{array} \end{array} \end{array} \end{array} \end{array} \end{array}\right],$$

and $I^{*}$ is the identity matrix with size nτ×nτ. The test statistic $F_{4}$ given by Eq. (41) follows F distribution, namely $F_{4}∼F_{\left(df1;df2\right)},$ where $df_{1}=\left(n\tau -p-1\right)$, and $df_{2}=\frac{\delta_{1}^{2}}{\delta_{2}},$ with $\delta_{1}=tr\left({\left(I^{*}-L^{*}\right)}^{T}\left(I^{*}-L^{*}\right)\right)$, and $\delta_{2}=tr{\left({\left(I^{*}-L^{*}\right)}^{T}\left(I^{*}-L^{*}\right)\right)}^{2}$. The critical region for similarity testing is to reject $H_{0}$ at the significance levelα if and only if $F_{4}>F_{\left(1-\alpha ;db1;db2\right)}$, or to reject $H_{0}$ if and only if $p_{7}<\alpha$ [11] with

(48) $$p_{7}=P\left(F_{v}>F_{4}\right)$$

*10. Partial Testing of the GWPR Model Parameters*

Partial testing useful to analyze the effect every predictor variable to the response variable. The hypothesis for *testing parameter* $\beta_{k}\left(u_{i}.v_{i}\right)$ *with the certain* i(i=1,2,…,n)*, and* k(k=1,2,…,p) *is*

$H_{0}$: $\beta_{k}\left(u_{i}.v_{i}\right)=0$ (The k-th predictor variable doesn’t affect the response variable)

$H_{1}$: $\beta_{k}\left(u_{i}.v_{i}\right)\neq 0\;$(The k-th predictor variable affects the response variable)

The test statistics is given by

(49) $$T_{3}=\frac{{\hat{\beta }}_{k}\;\left(u_{i}.v_{i}\right)}{\hat{\sigma }\sqrt{c_{kk}}},$$

with $c_{kk}$ is the k-th diagonal element of the $C^{T}C$ matrix, with $C={\left({X^{*}}^{T}W\left(u_{i}.v_{i}\right)X\right)}^{-1}{X^{*}}^{T}W\left(u_{i}.v_{i}\right)$ and

$\hat{\sigma }=\sqrt{\frac{y^{{*}^{T}}{\left(I^{*}-L^{*}\right)}^{T}\left(I^{*}-L^{*}\right)y^{*}}{tr({\left(I^{*}-L^{*}\right)}^{T}\left(I^{*}-L^{*}\right))}}$ [35]. The test statistic $T_{3}$ given by Eq. (47) follows t distribution, namely $T_{3}∼t_{\left(\frac{\delta_{1}^{2}}{\delta_{2}}\right)}$. The critical region for partial testing is to reject $H_{0}$ at the significance levelα if and only if $|T_{3}\;|>t_{\left(1-\frac{\alpha }{2};\frac{\delta_{1}^{2}}{\delta_{2}}\right)}$, or to reject $H_{0}$ if and only if $p_{8}<\alpha$ [35] with

(50) $$p_{8}=2P\left(T>\left|T_{3}\right|\right).$$

*11. The Measure of the Model Goodness-of-fit*

The measure of the model goodness-of-fit in this research is the coefficient of determination (R^2^), and root mean square error (RMSE) [34]. The coefficient of determination is given by

(51) $$R^{2}=\frac{\sum_{i=1}^{n}\sum_{t=1}^{\tau }{\left({\hat{y}}_{it}^{*}-{\bar{y}}^{*}\right)}^{2}}{\sum_{i=1}^{n}\sum_{t=1}^{\tau }{\left(y_{it}^{*}-{\hat{y}}_{it}^{*}\right)}^{2}},$$

and RMSE value is given by

(52) $$RMSE=\sqrt{\frac{1}{n\tau }\sum_{i=1}^{n}\sum_{t=1}^{\tau }{\left(y_{it}^{*}-{\hat{y}}_{it}^{*}\right)}^{2},}$$

with n is the number of sample size, and τ is the number of time period [27].

*12. Biochemical Oxygen Demand*

The biochemical oxygen demand (BOD) is the amount of oxygen consumed by microorganisms to decompose organic matter in water. The high BOD indicates the water quality declines. The Class 1 river water quality standard for drinking water source is displayed in Table 1, following Government Regulation No 22 of 2021 concerning the Implementation of Environmental Protection and Management [36].

**Table 1:** The Class 1 River Water Quality Standard.

BOD Level	Indication
≤2mg/L	*Good Quality*
>2mg/L	*Degraded Quality*

*13. Data Analysis Workflow*

This research employs descriptive statistics, the Fixed Effect Model (FEM), and Geographically Weighted Panel Regression (GWPR) to analyze the influencing factors of BOD in the Mahakam River Basin during 2022–2024. The workflow is summarized as *follows.*

*a. Descriptive Statistics*

Descriptive analysis of all variables, including the minimum, maximum, mean, and standard deviation, is performed in RStudio (R 4.3.1), while spatial distributions are visualized in QGIS (3.32.3) under the UTM Zone 50S projection.

*b. Data Transformation*

Panel data are transformed into demean (within) form using the plm package to remove time-invariant effects across locations.

*c. Multicollinearity Detection*

Multicollinearity is examined using the Variance Inflation Factor (VIF), where variables with VIF > 10 are re-evaluated before estimation.

*d. Fixed Effect Model (FEM)*

The FEM is estimated using the plm package. Diagnostic tests include the Glejser test for homoscedasticity, Jarque–Bera for normality, and Global Moran’s I for autocorrelation.

*e. Geographically Weighted Panel Regression (GWPR)*

GWPR estimation is conducted in GNU Octave version 9.3.0, in which the spatial weights are computed using an adaptive bi-square kernel. The Coordinate Reference System (CRS) used is geographic, represented by latitude–longitude pairs in degrees, and distances between observation points are calculated using the Euclidean distance after transforming all coordinates into a projected CRS with metric units (meters) using Google Earth. These metric distances are then used as inputs in the weighting function. The optimal bandwidth is determined using the golden-section algorithm by minimizing the AIC, and error diagnostics are performed using the same tests as in linear regression model.

*f. Mapping and Model Evaluation*

Spatial maps of significant factors are created in QGIS, and model performance is compared using R² and RMSE, where the model with higher R² and lower RMSE is considered superior.

*g. Software*

FEM analysis was performed in RStudio (R version 4.3.1) using the plm, car, and spdep packages, while GWPR model computations were conducted in GNU Octave (version 9.3.0). Spatial visualization and mapping were completed in QGIS (version 3.32.3).

## Method validation

*1. Research Data Description*

The research data is secondary data, namely the Analyze Report of the Surface Water Quality Monitoring from Life Environmental Department of the East Kalimantan Province 2022–2024. The sample size was 16 observation points with 48 observation data within 3 periods in 2022–2024. The research data consists of response variable data, namely BOD (y), and predictor variable data consisting of the data of water temperature $\left(x_{1}\right)$, water pH $\left(x_{2}\right)$, water color degree $\left(x_{3}\right)$, nitrate concentration $\left(x_{4}\right)$, ammonia concentration $\left(x_{5}\right)$, total suspended solid or TSS $\left(x_{6}\right)$, sulfate concentration $\left(x_{7}\right)$, also the coordinate data of observation location point. The description of the research data is stated into descriptive statistic consisting of the average, minimum value, maximum value, and standard deviation, which can be seen in Table 2.

**Table 2:** Descriptive Statistics of Reseach Data.

Variable	Year	Average	Minimum	Maximum	Standard Deviation	Water Quality Standard
BOD (y)	2022 2023	8.3060 7.8440	7.0000 7.0000	10.0000	0.9350	≤2mg/L
				9.0000	0.6250	
	2024	4.5870	2.0000	6.8950	1.2410	
Temperature $\left(x_{1}\right)$	2022	27.8400	26.0000	30.5000	1.2740	$\text{dev}\;3{░}^{\circ }C$ (*)
	2023	27.1600	25.0000	29.5000	1.7000	
	2024	28.5600	26.0000	30.0000	1.2890	
Water pH $\left(x_{2}\right)$	2022	7.2820	6.5550	9.0000	0.5680	6−9
	2023	7.2190	5.2800	7.8900	0.6330	
	2024	6.9710	4.3600	7.6600	1.0080	
Color Degree $\left(x_{3}\right)$	2022	127.3400	28.5000	192.0000	51.3650	≤15Pt−Co
	2023	240.7000	40.5000	827.0000	224.6000	
	2024	53.4100	9.0000	98.5000	26.4800	
Nitrate Concentration $\left(x_{4}\right)$	2022	0.2500	0.0200	0.9000	0.2580	≤10mg/L
	2023	1.8660	0.0900	5.0000	1.4170	
	2024	2.8450	1.0000	6.0000	1.3740	
Ammonia Concentration $\left(x_{5}\right)$	2022	0.1540	0.1000	0.6000	0.1790	≤0.1mg/L
	2023	0.7540	0.0750	9.0000	2.2010	
	2024	0.0840	0.0450	0.1500	0.0270	
TSS $\left(x_{6}\right)$	2022	154.4100	26.0000	467.0000	111.6720	≤40mg/L
	2023	187.8400	61.5000	479.0000	143.7870	
	2024	48.2800	16.5000	102.5000	22.4030	
Sulfate Concentration $\left(x_{7}\right)$	2022	18.2200	2.0200	164.0000	43.4260	≤300mg/L
	2023	17.2690	2.8800	115.0000	37.7670	
	2024	27.3000	6.9400	105.5000	30.5940	

(*): The maximum difference between the water and air temperatures measured at the water surface is 3 °C.

Based on Table 2, the BOD average of the Mahakam River in 2022, 2023, and 2024 respectively is 8.3060mg/L,7.8440mg/L,and4.5870mg/L, with the average of the maximum deviation respectively is 0.9350,0.6250,and1.2410. The river water BOD in 2022 to 2024 in the range of 2.0000−10.0000mg/L which more than standard quality (2mg/L) which indicates the Mahakam River water quality declines [36].

**Fig. 1:**
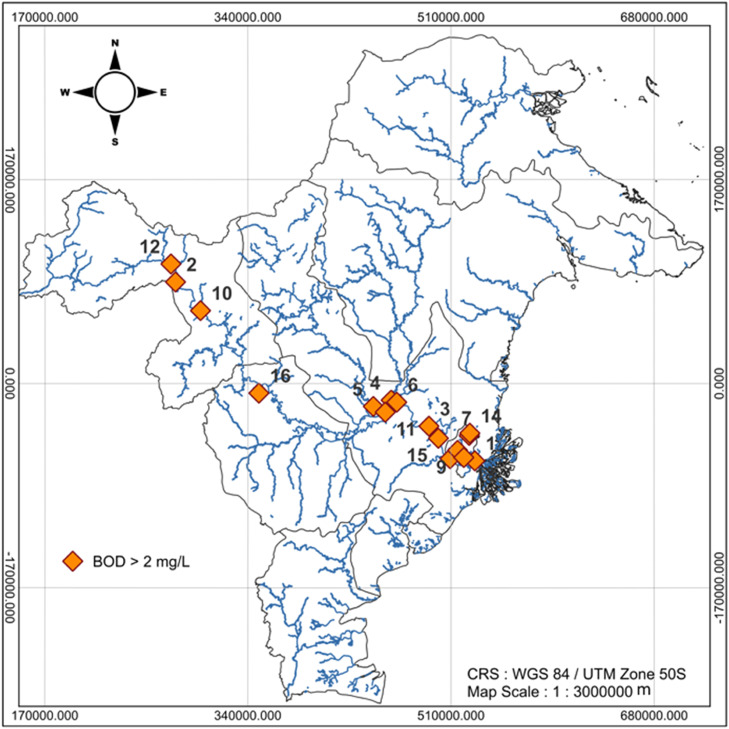
The BOD Level Distribution Map of the Mahakam River Mater in 2022.

**Fig. 2:**
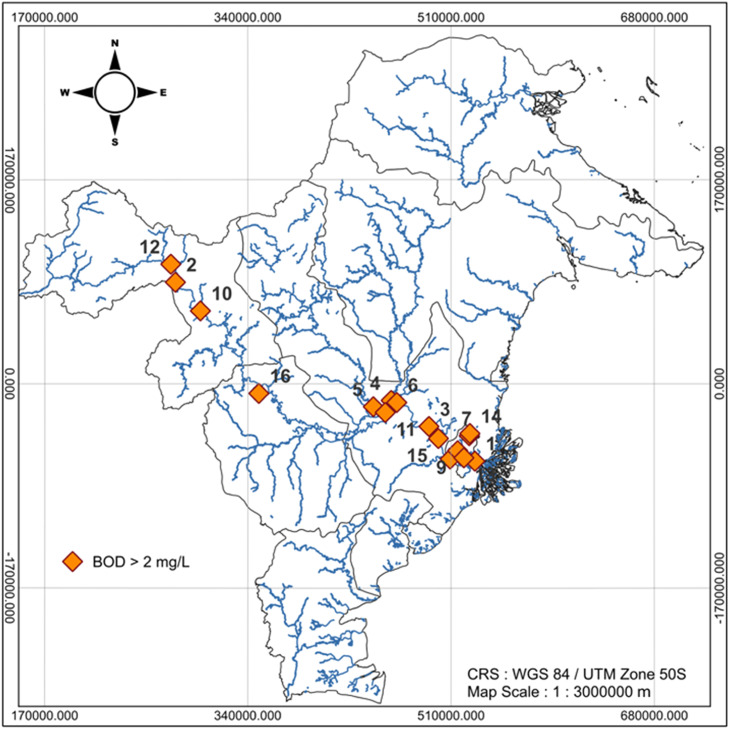
The BOD Level Distribution Map of the Mahakam River Water in 2023.

**Fig. 3:**
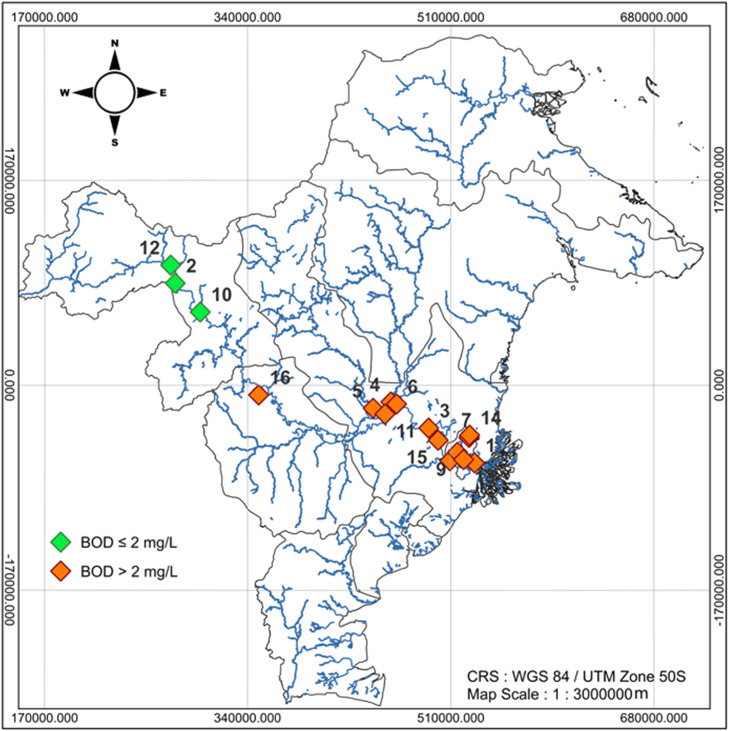
The BOD Level Distribution Map of the Mahakam River Water in 2024.

The description of the Mahakam River BOD in this research is also visualized through the BOD distribution at each observation location displayed in Fig. 1, Fig. 2, and Fig. 3. Fig. 1, Fig. 2, and Fig. 3 are the map of the East Kalimantan Province area, and the blue curves (lines) are river distributed in Kalimantan Province area. The dots consisting of the orange dot and green dot represent the observation location point. The orange dots in Fig. 1, Fig. 2, and Fig. 3 state the observation location points of the Mahakam River water in 2022–2024 with BOD is more than 2mg/L which indicates the quality of the Mahakam River water decline. While, the green dots in Fig. 3 represent the observation location point of the Mahakam River water, in 2024 with BOD is less than 2mg/L indicating the Mahakam River water quality is good.

The BOD mapping of Mahakam River water in 2022, 2023, 2024 respectively can be seen in Fig. 1, Fig. 2, and Fig. 3

Fig. 1, Fig. 2, and Fig. 3 illustrate the spatial distribution of BOD concentrations in the Mahakam River, Kalimantan, for the years 2022, 2023, and 2024, respectively. The maps were generated using QGIS (version 3.32.3) with a projected Coordinate Reference System (CRS) of WGS 84 / UTM Zone 50S and a map scale of 1:3.000.000 m.

Based on Fig. 1, that BOD at all observation location points in 2022 is more than 2mg/L which indicates the Mahakam River Water quality in 2022 decreases. It is shown by all of observation location points marked by orange dots in the Fig. 1. Based on Fig. 2, all of observation location points (16 points) are represented by orange dot which indicate the Mahakam River water quality in 2023 decreases, namely their BOD are more than 2mg/L.

It can be seen in Fig. 3, that the Mahakam River water at 81.25% of observation location points or 13 out of 16 observation location points in 2024 is marked by orange dot with their BOD are more than 2mg/L, which indicates the Mahakam River water quality decreases. While, the Mahakam River water BOD at 18.75% of observation location points or 3 out of 16 observation points in 2024 respectively is less than 2mg/L which indicates the Mahakam River water quality is good. It is shown by the observation location points marked by green dot in the Fig. 3.

**Fig. 4:**
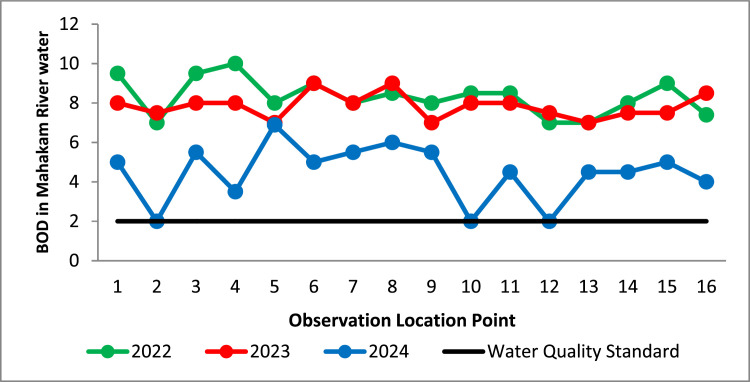
The BOD Scatter Plot of the Mahakam River Water BOD in 2022–2024.

The descriptive statistic of the Mahakam River BOD in 2022–2024 in this research is visualized through scatter plot displayed in Fig. 4. Fig. 4 is the scatter plot of BOD of the Mahakam River water at 16 observation points during 2022, 2023, and 2024. The black line in Fig. 4 shown the Class 1 of river water quality standard, namely is 2mg/L. The red, blue, and green scatter plot in Fig. 4 respectively represents the BOD observation results in 2022, 2023, and 2024.

It can be seen in Fig. 4, the scatter plot of the Mahakam River water BOD in 2022 (green line), and 2023 (red line) respectively is a above the black line. It shows BOD in all observation location points are more than 2mg/L, which indicates the Mahakam River water quality in 2022, and 2023 decrease. Based on scatter plot BOD in 2024 displayed by Fig. 4, the Mahakam River water BOD at 3 observation points (2-th, 10-th, and 12-th observation point) is conform to quality standard (≤2mg/L). Meanwhile, the Mahakam River water BOD in 2024 at 13 observation location are above the black line which show the Mahakam River water at 13 observation location points decrease. Generally, based on the BOD description that the Mahakam River water quality in 2022–2024 decreases.

It can be seen in Table 2, the river water temperature of Mahakam River water in 2022–2024 in the range of $25-30.5{\;}^{\circ }C$. The water temperature average of Mahakam River water in 2022–2024 respectively is 840 °C., 27.160 °C, and 28.5600 °C with the maximum deviation respectively is 1.2740, 1.7000, and 1.2890. The Mahakam River water temperature based on observation result in 2022–2024 it matches the standard quality, namely 25–30 °C.. The pH of the Mahakam River water in 2022–2024 in the range 4.360–9.000. The pH average in 2022–2024 respectively is 7.2820, 7.2190, and 6.9710, with the maximum deviation respectively is 0.5680, 0.6330, and 1.0080. These results indicate that the pH level of the Mahakam River water was still within the quality standard, namely in the range of 6–9. The water color degree of Mahakam River during 2022–2024 in the range of 50.000–325.000 Pt-Co. The average of water color degree in 2022, 2023, and 2024 respectively is 127.5630 Pt-Co, 147.0630 Pt-Co, and 118.6250 PtCo, with the maximum deviation of 59.2980, 81.7650, and 61.6120 respectively. These values exceeded the water quality standard of 15 Pt-Co indicating the Mahakam River water during the observation period was consistently above the permissible limit for color.The nitrate concentration of Mahakam River water during 2022–2024 ranged from 0.0630 to 1.2450 mg/L. The average of nitrate concentration in 2022, 2023, and 2024 respectively is 0.3790 mg/L, 0.3920 mg/L, and 0.5560 mg/L, with maximum deviation of 0.2480, 0.2590, and 0.3380 respectively. These results indicate that nitrate concentrations of the Mahakam River water in 2022–2024 were below the quality standard threshold of 10 mg/L meaning that during the research period the nitrate levels were within acceptable limits. The ammonia concentration during 2022–2024 ranged 0.0080–0.4210 mg/L. The average of ammonia concentration in 2022, 2023, and 2024 respectively is 0.1330 mg/L, 0.1540 mg/L, and 0.117 mg/L, with maximum deviations of 0.1220, 0.1480, and 0.1080 respectively. These values were below the water quality standard of 0.5 mg/L, which indicates that the concentration of ammonia in the Mahakam River water to reached the standards accepted throughout the year of observation. The Total Suspended Solid (TSS) of Mahakam River water during 2022–2024 ranged 1.0000–158.0000 mg/L. The average of TSS in 2022, 2023, and 2024 respectively is 54.0000 mg/L, 47.6250 mg/L, and 28.1880 mg/L, with maximum deviations of 51.459, 52.508, and 24.043 respectively. These observations show that TSS exceeded the water quality standard of 50 mg/L in several observation points, particularly in 2022 and 2023, while in 2024 the TSS generally conformed to the standard. And the sulfate concentration during 2022–2024 ranged 1.0000–23.0000 mg/L. The average of sulfate concentration in 2022, 2023, and 2024 respectively is 9.2500 mg/L, 10.1880 mg/L, and 6.4380 mg/L, with maximum deviations of 6.9750, 8.370, and 3.8270 respectively. These results indicate that sulfate concentrations of the Mahakam River water were consistently below the water quality standard of 400 mg/L, which indicates that the sulfate related water quality is well below the acceptable limit.

*2. Multicollinearity Detection Between Predictor Variable*

Multicollinearity detection between predictor variable in this research uses VIF criterion. Based on the computation result of VIF referring to Eq. (35) using R software, the VIF value for all predictor variables are displayed in Table 3.

**Table 3:** VIF Value.

*Predictor Variable*	*VIF Value*
*Temperature*$\left(x_{1}\right)$	1.1410
*Water* pH $\left(x_{2}\right)$	1.7322
*Color Degree*$\left(x_{3}\right)$	3.2655
*Nitrate Concentration*$\left(x_{4}\right)$	1.4220
*Ammonia Concentration*$\left(x_{5}\right)$	1.0332
*TSS*$\left(x_{6}\right)$	3.3271
*Sulfate Concentration*$\left(x_{7}\right)$	1.6273

Based on Table 3, the VIF value of every predictor variable respectively is <10, and it is concluded there is no multicollinearity between the predictor variables, and therefore FEM modeling can involve 7 predictor variables presented in Table 3.

*3. FEM Model*

The general model of FEM within estimator that expresses the relationship between the BOD (y) of Mahakam River water and 7 predictor variabless displayed in Table 3 is

(53) $$y_{it}^{*}=\beta_{1}x_{it1}^{*}+\beta_{2}x_{it2}^{*}+\beta_{3}x_{it3}^{*}\ldots +\beta_{7}x_{it7}^{*}+\varepsilon_{it}^{*},;i=1,2,\ldots ,16;\;t=1,2,3.$$

Parameter estimation of FEM given by Eq. (53) is OLS referring to Eq. (18). Computation of parameter estimation uses R software, and its result can be seen in Table 4.

(54) $$y_{it}^{*}=-0.46621x_{it1}^{*}-1.06702x_{it2}^{*}+0.00428x_{it3}^{*}-1.02443x_{it4}^{*}+0.23125x_{it5}^{*}+0.00053x_{it6}^{*}+0.00477x_{it7}^{*};\;i=1,2,\ldots ,16;\;t=1,2,3$$

**Table 4:** Parameter Estimator of FEM Model.

Parameter	Estimation
$\beta_{1}$	−0.46621
$\beta_{2}$	−1.06702
$\beta_{3}$	0.00428
$\beta_{4}$	−1.02443
$\beta_{5}$	0.23125
$\beta_{6}$	0.00053
$\beta_{7}$	0.00477

Based on Table 4, the FEM (the global model) has an expression.

*4. Significance Testing of FEM Parameters*

The significance test of the parameter of FEM consists of the simultaneous testing and partial testing. The first significance test is the simultaneous testing, with the hypothesis is.

$H_{0}$: $\beta_{1}=\beta_{2}=\beta_{3}=\beta_{4}=\beta_{5}=\beta_{6}=\beta_{7}=0$ (the FEM is not fit).
$H_{1}$: At least one $\beta_{k}\neq 0;\;k=1,2,3,4,5,6,7$ (the FEM is fit).

The test statistic is given by Eq. (20), and the calculation results of the simultaneous test are presented in Table 5

**Table 5:** Test Statistic Values on the Simultaneous Test of FEM Parameters.

	$F_{\left(0,95;7;25\right)}$	$p_{3}$	Decision
9.0712	2.4047	$1.4815\;\times 10^{-5}$	$H_{0}$ is Rejected

Based on Table 5, the test decision is to reject $H_{0}$ at the significance level of 0.05. The simultaneous test concludes the FEM is fit model or all predictor variables $x_{1},\;x_{2},\;\;x_{3},\;\;x_{4},x_{5},x_{6},\;and\;x_{7}$ simultaneously affect to the Mahakam River water BOD. It is confirmed by the statistic value of $F_{2}=9.0712>F_{\left(0,95;7;25\right)}=2.0712$, and $p_{3}=1.4815\;\times 10^{-5}<\alpha =0.05$.

The second significance test is the partial testing. The hypothesis for testing parameter $\beta_{k}$ with the certain k (k=1,2,…,p) is

$H_{0}$: $\beta_{k}=0$ (There is no effect of the predictor variable $x_{k}$ on the BOD of the Mahakam River Water)
$H_{1}$: $\beta_{k}\neq 0$ (There is an influence of the predictor variable $x_{k}$ on the BOD of the Mahakam River Water)

The test statistic is given by Eq. (24), and the resume of calculation results of partial significance testing is presented in Table 6.

**Table 6:** The Statistic Values of Partial Testing.

Variable	$|T_{2}|$	$p_{4}$	*Decision*
*Temperature*$\left(x_{1}\right)$	1.5794	0.1268	$H_{0}$ is Accepted
*Water* pH $\left(x_{2}\right)$	2.1829	0.0386	$H_{0}$ is Rejected
*Water Color Degree*$\left(x_{3}\right)$	1.4552	0.1581	$H_{0}$ is Accepted
*Nitrate Concentration*$\left(x_{4}\right)$	5.5355	0.0000	$H_{0}$ is Rejected
*Ammonia Concentration*$\left(x_{5}\right)$	1.2198	0.2339	$H_{0}$ is Accepted
*TSS*$\left(x_{6}\right)$	0.1262	0.9006	$H_{0}$ is Accepted
*Sulfate Concentration*$\left(x_{7}\right)$	0.2745	0.7860	$H_{0}$ is Accepted

Test statistic $T_{2}$ displayed in Table 6 follows t distribution, namely $T_{2}\;∼t_{\left(41\right)}$, and using the significance level of 0.05 the critical value for partial test is $t_{\left(0,975;41\right)}=\;2,0211$. Based on test statistic values displayed on Table 6, the partial test to reject $H_{0}$ for predictor variables water pH ($x_{2}$,) and nitrate concentration$\;(x_{4})$. It is confirmed by the statistic of $|T_{2}|$ for water pH ($x_{2}$,) and nitrate concentration$\;(x_{4})$ respectively is more than the critical value $t_{\left(0,975;41\right)}=\;2,0211$, and p-value ($p_{4}$) for two variables respectively is less than the significance level of 0.05. Therefore, the predictor variables water pH ($x_{2}$,) and nitrate concentration$\;(x_{4})$ respectively affects individually to Mahakam River water BOD. Meanwhile, $|T_{2}|$ values for variables temperature $\left(x_{1}\right)$, color degree $\left(x_{3}\right)$, ammonia concentration $\left(x_{5}\right)$, TSS $\left(x_{6}\right)$, and sulfate concentration $\left(x_{7}\right)$ respectively is less than the critical value $t_{\left(0,075;41\right)}=\;2,0211$, and concludes their p-value ($p_{4}$) respectively is more than 0.05. These test statistic values recommend to accept $H_{0}$ for variable temperature $\left(x_{1}\right)$, color degree $\left(x_{3}\right)$, ammonia concentration $\left(x_{5}\right)$, TSS $\left(x_{6}\right)$, sulfate concentration $\left(x_{7}\right)$, and concludes they do not affect individually to Mahakam River water BOD.

*5. Error Homoscedasticity Testing of the FEM Model*

The first assumption test for the FEM model is error homoscedasticity testing. Homoscedasticity testing in the research uses the Glejser approach. Homoscedasticity testing also useful to test the spatial heterogeneity data of response variable (BOD). The hypothesis of the error homoscedasticity testing is

$H_{0}$: $\sigma_{11}^{2}=\sigma_{2t}^{2}=\ldots =\sigma_{16t}^{2}=\sigma^{2};t=1,2,3$
(The variance error is constant or the response variable data is not spatial heterogeneity data)
$H_{1}$: at least one $\sigma_{it}^{2}\neq \sigma^{2};i=1,2,\ldots ,\;16;t=1,2,3$
(The variance error does not constant or the he response variable data is spatial heterogeneity data)

The test statistic is given by Eq. (27), and the calculation results of homoscedasticity testing are presented in Table 7.

**Table 7:** Glejser Test Results for FEM Model.

	$F_{\left(0,95;8;24\right)}$	$p_{5}$	Decision
2.5354	2.7141	0.0371	$H_{0}$ is Rejected

Based on Table 7, $H_{0}$ is rejected at the significance level of 0.05, and it can be concluded that the variance of error does not constants or it is equivalent to variance of response variable (BOD) does not constant. Its mean the response variable data is spatial heterogeneity data, namely BOD data was heterogeneity spatial data. This decision is confirmed by $F_{3}=2.5354>F_{\left(0,95;8;24\right)}=2.7141$, and $p_{5}=0,0371<\alpha =0,05$. Based on the homoscedasticity testing, it confirms the FEM doesn’t satisfy the assumption of classical linear regression, and therefore the FEM is not fit. Since the homoscedasticity assumption of FEM error was not satisfy, checking for other assumption such as the error normality, and the absence of autocorrelation among errors was deemed unnecessary. Meanwhile, based on the spatial heterogeneity data testing for BOD data was confirmed as heterogeneity spatial data, therefore the GWPR modeling to the Mahakam River water BOD data can be recommended.

*GWPR Modeling on the Data of Makam River Water BOD*

The general GWPR model at the i th location and the t-th time, referring to Eq. (36) and describing the relationship between the Mahakam River water BOD and 7 predictor variables displayed in Table 3, is expressed as follows:

(55) $$y_{it}^{*}=\beta_{1}\left(u_{i},v_{i}\right)x_{it1}^{*}+\beta_{2}\left(u_{i},v_{i}\right)x_{it2}^{*}+\ldots +\beta_{7}\left(u_{i},v_{i}\right)x_{it7}^{*}+\varepsilon_{it}^{*};\;i=1,2,\ldots ,16;\;t=1,2,3.$$

The Parameter estimation is carried out at each observation location using the weighted least squares (WLS) method referring to Eq. (41), applied to the demeaned data as defined in Eq. (16). All spatial data were reprojected into a projected coordinate reference system (UTM Zone 50S), and all inter-site distances were computed consistently in metric units based on these projected coordinates, with distance verification conducted using Google Earth. These distances are subsequently used to determine the optimal adaptive bandwidth by minimizing the Akaike Information Criterion (AIC) referring to Eq. (38). The optimal bandwidth for the adaptive bi-square spatial weighting function (Eq. (36)) is obtained through a golden-section search algorithm implemented in GNU Octave 9.3.0. The search is performed over a feasible range of bandwidth values and iteratively refined until no further improvement in the AIC value is achieved, ensuring a stable and efficient optimal bandwidth for each observation location.

Table 8.

**Table 8:** Optimum Bandwidth and AIC Values for Each Observation Location.

i	Best Bandwidth	AIC
1	91,368.6528	−7.4768
2	82,133.6149	−60.1510
3	225,488.8356	−264.1756
4	83,312.2914	−131.3828
5	279,674.5927	−12.9843
6	83,316.8708	22.4211
7	268,588.8101	−17.2644
8	302,770.7384	−115.7725
9	145,800.2343	−115.6276
10	302,770.7384	−45.9929
11	177,597.8699	−45.2475
12	302,770.7384	−57.2117
13	0.0025	−43.0294
i	Best Bandwidth	AIC
14	302,770.7384	−100.9136
15	96,732.3810	20.8719
16	203,985.0754	3.6660

All geographically weighted computations, including the construction of local weighting matrices, bandwidth optimization, and parameter estimation, are performed in GNU Octave 9.3.0. The fixed effects estimation results at each observation location constitute the GWPR model, representing the local average BOD behavior of the Mahakam River. In total, 16 local FEM-based GWPR models are obtained, and the corresponding parameter estimates are presented in Table 9.

(56) $$y_{1t}^{*}=1.1297x_{\text{it}1}^{*}-2.7082x_{\text{it}2}^{*}+0.0229{\;x}_{\text{it}3}^{*}-0.4970x_{\text{it}4}^{*}+0.2587x_{\text{it}5}^{*}+0.0157x_{\text{it}6}^{*}+0.0181x_{\text{it}7}^{*};\;t=1,2,3.$$

**Table 9:** Parameter Estimator of GWPR Model at 16 Observation Points along the Mahakam River.

i	Observation Point	$\beta_{1}\left(u_{i},v_{i}\right)$	$\beta_{2}\left(u_{i},v_{i}\right)$	$\beta_{3}\left(u_{i},v_{i}\right)$	$\beta_{4}\left(u_{i},v_{i}\right)$	$\beta_{5}\left(u_{i},v_{i}\right)$	$\beta_{6}\left(u_{i},v_{i}\right)$	$\beta_{7}\left(u_{i},v_{i}\right)$
1	Anggana	1.1297	−2.7082	0.0229	−0.4970	0.2587	0.0157	0.0181
2	Batoq Kelo	0.4206	−1.4850	0.0003	−1.2795	0.4635	0.0069	0.4541
3	Bloro	0.9354	−3.1176	−0.0097	−0.4654	0.3359	0.0219	0.0087
4	Kedang Kepala Village	0.0516	−3.3592	−0.0069	−0.6619	0.6665	0.0245	0.0396
5	Sebelimbingan Village	1.1072	−3.3047	−0.0097	−0.6577	0.3702	0.0212	0.0145
6	Siran Village	0.0997	−3.3512	−0.0069	−0.6537	0.4317	0.0237	0.0332
7	Pampang Village Bridge	0.9514	−3.0717	−0.0093	−0.4677	0.3326	0.0211	0.0069
8	Kalamur	1.0028	−3.1275	−0.0097	−0.4919	0.3414	0.0215	0.0086
9	East Kalimantan Province Governor's Office	1.1297	−2.7082	0.0229	−0.4970	0.2587	0.0157	0.0181
10	Long Bagun	0.9102	−2.9166	−0.0060	−0.5449	0.3192	0.0163	0.0053
11	Muara Sungai Belayan	1.4801	−4.2531	−0.0081	−1.4144	0.5964	0.0188	0.0473
12	Nyan Mahulu	0.9028	−3.2450	0.0107	0.4625	0.3470	0.0238	0.0137
13	Palaran	1.3921	−4.6836	0.0086	1.7219	2.9953	0.0203	0.0825
14	Pampang Dalam	0.0015	0.0009	0.0280	0.0015	0.0130	0.0066	0.0013
15	Pulau Kumala	0.9868	−3.1131	−0.0095	0.4894	0.3388	0.0213	0.0079
16	Tering	0.8858	−2.9966	−0.0045	0.6173	0.3245	0.0144	0.0102

Based on Table 9, for example, GWPR model at the Anggana point (the 1-th observation point) is expressed as follows:.

The model presented in Eq. (56) can be expressed as follows:

(57) $$\left(y_{1t}-{\bar{y}}_{1}\right)=1.1297\left(x_{1t1}-{\bar{x}}_{11}\right)-2.7082\left(x_{1t2}-{\bar{x}}_{12}\right)+0.0229\left(x_{1t3}-{\bar{x}}_{13}\right)-0.4970\left(x_{1t4}-{\bar{x}}_{14}\right)+0.2587\left(x_{1t5}-{\bar{x}}_{15}\right)+0.0157\left(x_{1t6}-{\bar{x}}_{16}\right)+0.0181\left(x_{1t7}-{\bar{x}}_{17}\right)$$

Applying the demeaned data to model given by Eq. (57), and referring to Eq. (12), yields

(58) $$\left(y_{1t}-7.5000\right)=1.1297\left(x_{1t1}-28.6667\right)-2.7082\left(x_{1t2}-7.5350\right)+0.0229\left(x_{1t3}-126.5000\right)-0.4970\left(x_{1t4}-1.0233\right)+0.2587\left(x_{1t5}-0.0783\right)+0.0157\left(x_{1t6}-70.5000\right)+0.0181\left(x_{1t7}-6.9483\right),$$

and after simplifying Eq. (58), it is obtained the GWPR model at the observation point at Anggana as follows:

(59) $${\hat{y}}_{1t}=-8.1196+1.1297x_{1t1}-2.7082x_{1t2}+0.0229\;x_{1t3}-0.4970x_{1t4}+0.2587x_{1t5}+0.0157x_{1t6}\;+0.0181x_{1t7};\;t=1,2,3.$$

Referring to Table 9, and Eq. (59) a total of 16 GWPR models are obtained. The GWPR model at every observation point is different, and the model for each time period in one location is same.

*7. Similarity Testing between GWPR and FEM Models*

The hypothesis of similarity testing between GWPR and FEM models is

$H_{0}$: $\beta_{k}\left(u_{i}.v_{i}\right)=\beta_{k},\;i=1,\;2,\;\ldots ,\;16;\;k=1,2,3,4,5,6,7$ (The FEM and GWPR models are similar)
$H_{1}$: there is at least one $\beta_{k}\left(u_{i}.v_{i}\right)\neq \beta_{k},\;i=1,\;2,\;\ldots ,\;16;k=1,\;2,3,4,5,6,7$ (The FEM and GWPR models are not similar)

The similarity test statistic is given by Eq. (43), and the calculation results are summarized in Table 10.

**Table 10:** Similarity Testing of GWPR and FEM Models.

	$F_{\left(0.95;27;40\right)}$	$p_{7}$	Decision
2.2530	1.7260	0.0074	$H_{0}$ is Rejected

It can be seen in Table 10, the test decision is to reject $H_{0}$ at the significance level of 0.05. The similarity test concludes the FEM and GWPR models are not similar, or the model is local model (GWPR). It is confirmed by the test statistic of $F_{4}=42.2530>F_{\left(0.05;27;40\right)}=1.7260$, and $p_{7}=0.0074<\alpha =0.05$.

*8. Partially GWPR Model Parameter Testing*

The hypothesis for testing parameter $\beta_{k}\left(u_{i}.v_{i}\right)$ with the certain i(i=1,2,…,16), and k(k=1,2,3,4,5,6,7) is

$H_{0}$: $\beta_{k}\left(u_{i}.v_{i}\right)=0$ (There is no influence of the k-th predictor variable to the BOD)
$H_{1}$: $\beta_{k}\left(u_{i}.v_{i}\right)\neq 0\;$(There is an influence of the k-th predictor variable to the BOD)

The test statistic is given by Eq. (49), and the results of the calculations for the GWPR models are summarized in Table 11.

**Table 11:** Partial Test Result of GWPR Model.

i	Test Statistics	Parameter						
		$\beta_{1}\left(u_{i},v_{i}\right)$	$\beta_{2}\left(u_{i},v_{i}\right)$	$\beta_{3}\left(u_{i},v_{i}\right)$	$\beta_{4}\left(u_{i},v_{i}\right)$	$\beta_{5}\left(u_{i},v_{i}\right)$	$\beta_{6}\left(u_{i},v_{i}\right)$	$\beta_{7}\left(u_{i},v_{i}\right)$
1	Estimation	1.1297	−2.7082	0.0229	−0.4970	0.2587	0.0157	0.0181
	Standard Error	0.6135	1.2298	0.0149	0.5403	0.2986	0.0185	0.0320
	$T_{3}$	1.8413	−2.2021	1.5321	−0.9199	0.8664	0.8464	0.5649
	$p_{8}$	0.0747	0.0348	0.1351	0.3644	0.3926	0.4035	0.5760
2	Estimation	0.4206	−1.4850	0.0003	−1.2795	0.4635	0.0069	0.4541
	Standard Error	2.5785	16.1603	0.2487	4.8262	0.2283	0.0771	4.1995
	$T_{3}$	0.1631	−0.0919	0.0011	−0.2651	2.0305	0.0896	0.1081
	$p_{8}$	0.8714	0.9273	0.9991	0.7926	0.0290	0.9292	0.9146
3	Estimation	0.9354	−3.1176	−0.0097	−0.4654	0.3359	0.0219	0.0087
	Standard Error	0.5074	0.8108	0.0051	0.3577	0.2925	0.0076	0.0266
	$T_{3}$	1.8434	−3.8452	−1.9190	−1.3008	1.1486	2.8896	0.3252
	$p_{8}$	0.0744	0.0005	0.0638	0.2024	0.2591	0.0068	0.7471
4	Estimation	0.0516	−3.3592	−0.0069	−0.6619	0.6665	0.0245	0.0396
	Standard Error	1.0401	1.2207	0.0071	0.4858	2.0073	0.0097	0.0501
	$T_{3}$	0.0496	−2.7519	−0.9783	−1.3626	0.3320	2.5356	0.7901
	$p_{8}$	0.9607	0.0096	0.3351	0.1823	0.7420	0.0162	0.4352
5	Estimation	1.1072	−3.3047	−0.0097	−0.6577	0.3702	0.0212	0.0145
	Standard Error	0.5001	0.7794	0.0048	0.3297	0.3129	0.0069	0.0278
	$T_{3}$	2.2137	−4.2402	−2.0203	−1.9945	1.1829	3.0548	0.5215
	$p_{8}$	0.0339	0.0002	0.0516	0.0545	0.2454	0.0045	0.6056
6	Estimation	0.0997	−3.3512	−0.0069	−0.6537	0.4317	0.0237	0.0332
	Standard Error	0.9621	1.1820	0.0067	0.4708	1.1612	0.0095	0.0446
	$T_{3}$	0.1036	−2.8352	−1.0302	−1.3884	0.3718	2.4935	0.7448
	$p_{8}$	0.9181	0.0078	0.3105	0.1744	0.7125	0.0179	0.4618
7	Estimation	0.9514	−3.0717	−0.0093	−0.4677	0.3326	0.0211	0.0069
	Standard Error	0.5018	0.8221	0.0052	0.3628	0.2853	0.0078	0.0265
	$T_{3}$	1.8959	−3.7362	−1.7876	−1.2891	1.1657	2.7097	0.2609
	$p_{8}$	0.0669	0.0007	0.0831	0.2064	0.2522	0.0106	0.7958
8	Estimation	1.0028	−3.1275	−0.0097	−0.4919	0.3414	0.0215	0.0086
	Standard Error	0.4923	0.7965	0.0050	0.3510	0.2841	0.0075	0.0264
	$T_{3}$	2.0371	−3.9266	−1.9209	−1.4014	1.2017	2.8717	0.3245
	$p_{8}$	0.0498	0.0004	0.0635	0.1705	0.2381	0.0071	0.7477
9	Estimation	0.9102	−2.9166	−0.0060	−0.5449	0.3192	0.0163	0.0053
	Standard Error	0.5271	0.9328	0.0060	0.3975	0.2858	0.0091	0.0272
	$T_{3}$	1.7269	−3.1267	−1.0048	−1.3708	1.1171	1.7839	0.1945
	$p_{8}$	0.0936	0.0037	0.3224	0.1798	0.2721	0.0837	0.8470
10	Estimation	1.4801	−4.2531	−0.0081	−1.4144	0.5964	0.0188	0.0473
	Standard Error	0.7733	1.1732	0.0066	0.4008	0.9099	0.0088	0.0618
	$T_{3}$	1.9139	−3.6252	−1.2215	−3.5288	0.6555	2.1362	0.7654
	$p_{8}$	0.0644	0.0010	0.2307	0.0013	0.5168	0.0403	0.4495
11	Estimation	0.9028	−3.2450	0.0107	0.4625	0.3470	0.0238	0.0137
	Standard Error	0.5703	0.8487	0.0052	0.3748	0.3458	0.0077	0.0295
	$T_{3}$	1.5831	−3.8236	2.0739	1.2339	1.0033	3.1080	0.4649
	$p_{8}$	0.1230	0.0006	0.0461	0.2260	0.3231	0.0039	0.6451
12	Estimation	1.3921	−4.6836	0.0086	1.7219	2.9953	0.0203	0.0825
	Standard Error	0.9684	1.5288	0.0087	0.5062	4.3951	0.0112	0.1311
	$T_{3}$	1.4375	−3.0635	0.9850	3.4014	0.6815	1.8090	0.6291
	$p_{8}$	0.1601	0.0044	0.3319	0.0018	0.5003	0.0797	0.5337
13	Estimation	0.0015	0.0009	0.0280	0.0015	0.0130	0.0066	0.0013
	Standard Error	0.0083	0.0048	0.0848	0.0088	0.0645	0.0898	0.0006
	$T_{3}$	0.1788	0.1856	0.3298	0.1724	0.2020	0.0732	2.1900
	$p_{8}$	0.8592	0.8539	0.7436	0.8642	0.8412	0.9421	0.0310
14	Estimation	0.9868	−3.1131	−0.0095	0.4894	0.3388	0.0213	0.0079
	Standard Error	0.4939	0.8036	0.0051	0.3540	0.2851	0.0076	0.0264
	$T_{3}$	1.9980	−3.8740	−1.8721	1.3828	1.1883	2.8185	0.2982
	$p_{8}$	0.0541	0.0005	0.0702	0.1761	0.2433	0.0081	0.7674
15	Estimation	0.8858	−2.9966	−0.0045	0.6173	0.3245	0.0144	0.0102
	Standard Error	0.5634	0.9946	0.0064	0.4183	0.3082	0.0098	0.0278
	$T_{3}$	1.5723	−3.0127	−0.6968	1.4758	1.0530	1.4710	0.3661
	$p_{8}$	0.1255	0.0050	0.4909	0.1496	0.3001	0.1509	0.7166
16	Estimation	1.7533	−4.2477	−0.0128	1.0934	0.7248	0.0244	0.0592
	Standard Error	0.8647	1.1383	0.0068	0.4653	1.2455	0.0089	0.0696
	$T_{3}$	2.0277	−3.7315	−1.8820	2.3500	0.5820	2.7457	0.8511
	$p_{8}$	0.0508	0.0007	0.0688	0.0250	0.5646	0.0097	0.4009

It can be seen in Table 11, that the $|T_{3}|$ value for variable water pH $\left(x_{2}\right)$ is 2.201 which is greater than the critical value $t_{\left(0,025;26,028\right)}=2,0414$, and also $p_{8}$ value for variable nitrate concentration $\left(x_{4}\right)$ is 0.0348, which is less than the significance level α=0.05. Therefore, the partial test recommends rejecting H₀ for this predictor variable, indicating that water pH significantly affects the BOD of the Mahakam River at the observation point in Anggana. Meanwhile, $|T_{3}|$ value for predictor variables temperature $\left(x_{1}\right)$, color degree $\left(x_{3}\right)$, nitrate concentration $\left(x_{4}\right)$, ammonia concentration $\left(x_{5}\right)$, TSS $\left(x_{6}\right)$, and sulfate concentration $\left(x_{7}\right)$ respectively is less than the critical value, and also $p_{8}$ respectively is less than significance value α=0.05. Therefore, the partial test recommends to accept $H_{0}$, and concludes that these variables don’t significantly affect the Mahakam River BOD at observation point in Anggana. Using the same way, the influencing factor of BOD at other observation locations can be obtained.

Based on Table 11, the GWPR produces different local models for each observation location point, and the model for each period in one location is same (identic). In this section, three models are presented as examples to show the estimation results and to interpretate of model parameter, namely the GWPR model at Anggana (at the 1-th), at Siran Village (at the 6-th), and at Tering (at the 16-th) observation points. The GWPR model at the Anggana observation location point based on the demeaned data is

(60) $${\hat{y}}_{1t}^{*}=1.1297x_{1t1}^{*}-2.7082x_{1t2}^{*}+0.0229x_{1t3}^{*}-0.4970x_{1t4}^{*}+0.2587x_{1t5}^{*}+0.0157x_{1t6}^{*}+0.0181x_{1t7}^{*};\;t=1,2,3.$$

The GWPR model in Eq. (58) can be written as a model including the intercept, as follows:

(61) $${\hat{y}}_{1t}=-8.1196+1.1297x_{1t1}-2.7082x_{1t2}+0.0229\;x_{1t3}-0.4970x_{1t4}+0.2587x_{1t5}+0.0157x_{1t6}+0.0181x_{1t7};\;t=1,2,3.$$

Based on the partial test results displayed in Table 11 (for *i* = 1), it was found that the factor influencing BOD at observation point is water pH. This indicates that the Mahakam River water at the Anggana observation point experiences variations in pH that significantly affect BOD levels. Changes in pH can alter the activity of decomposer bacteria in breaking down organic waste, such as plant material, animal remains, or household waste, thereby influencing oxygen consumption and consequently BOD. This finding is supported by the GWPR model regression parameter for water pH at the Anggana observation point, which is −2.7082. This implies that for every 1 unit increase in water pH at Anggana, the BOD is expected to decrease by 2.7082 mg/L. Furthermore, based on observational data, the water pH values over the three-year period (2022–2024) at this location were 7.685 in 2022, 7.405 in 2023, and 7.515 in 2024, with an average of 7.535, which is within the normal range for river water quality in the region.

The similar way, the GWPR model including the intercept at the Siran observation point is given by

(62) $${\hat{y}}_{6t}=26.1280+0.0997x_{6t1}-3.3512x_{6t2}-0.0069\;x_{6t3}-0.6537x_{6t4}+0.4317x_{6t5}+0.0237x_{6t6}+0.0332x_{6t7};t=1,2,3.$$

Based on Table 11, the factors influencing BOD at the 6-th observation point (Siran Village) are water pH $\left(x_{2\;}\right)$, and TSS $\left(x_{6\;}\right)$. The GWPR model regression coefficient for the water pH variable at the Siran observation point is –3.3512, which indicates that a one-unit increase in the pH level of the Mahakam River water at Siran will decrease the local BOD by 3.3512 mg/L. This finding suggests that as the water becomes slightly more alkaline, the activity of decomposer bacteria may decrease, resulting in lower oxygen consumption and consequently reducing BOD levels. Furthermore, based on observational data, the average pH level of the Mahakam River at the Siran observation point over the three-year period (2022–2024) was 7.1167, which is slightly lower than the regional three-year average of 7.1573.

Based on Table 11, TSS is also a significant factor influencing BOD at 6-th observation point (Siran Village). The GWPR model regression coefficient for the TSS variable $\left(x_{6\;}\right)$ at the Siran observation point is 0.0237, indicating that a 1 mg/L increase in TSS concentration in the Mahakam River at Siran will increase the local BOD by 0.0237 mg/L. This result suggests that TSS carries organic and inorganic particles that may enhance bacterial decomposition activity. As bacteria break down these materials, oxygen consumption increases, leading to higher BOD levels. In addition, observational data show that the average TSS concentration at the Siran location over the 2022–2024 period was 276.083 mg/L, which is relatively high and supports the observed influence of TSS on BOD.

The GWPR model at the Tering observation point is

(63) $${\hat{y}}_{16t}=8.5192+1.7533x_{16t1}-4.2477x_{16t2}-0.0128x_{16t3}+1.0934x_{16t4}+0.7248x_{16t5}+\;0.0244x_{16t6}+0.0592x_{16t7};\;t=1,2,3.$$

Based on the partial test results displayed in Table 11 (for *i* = 16), the variables that significantly affect the Mahakam River BOD at the Tering observation point are water temperature$\;(x_{1,})$, water pH$\;(x_{2,})$, nitrate concentration $\left(x_{4}\right)$. and TSS $\left(x_{6}\right)$. The regression coefficient for the temperature variable is 1.7533, indicating that an increase of 1 °C in water temperature is expected to increase the BOD level at Tering by 1.7533 mg/L. Elevated water temperature typically enhances the metabolic and decomposition activity of microorganisms, thereby increasing oxygen consumption and resulting in higher BOD values. The regression coefficient for pH is –4.2477, meaning that a one-unit increase in pH will reduce the BOD level at Tering by 4.2477 mg/L. This suggests that more alkaline conditions may inhibit the activity of decomposer bacteria, leading to lower oxygen consumption and consequently decreasing BOD levels. The nitrate concentration also shows a significant positive effect, with a regression coefficient of 1.0934. This implies that every 1 mg/L increase in nitrate concentration is associated with a 1.0934 mg/L increase in BOD. As a nutrient source, nitrate can stimulate microbial growth, thereby accelerating decomposition processes and increasing oxygen demand, which ultimately elevates BOD. Meanwhile, the regression coefficient for TSS concentration is 0.0244, indicating that a 1 mg/L increase in TSS will increase BOD at the Tering observation point by 0.0244 mg/L. Higher TSS levels typically reflect the presence of suspended particles that often contain organic matter. As these materials undergo decomposition, they contribute to higher oxygen consumption, leading to increased BOD levels.

Based on the affecting factors the Mahakam River water BOD based on partial test displayed in Table 11, it can be obtained the affecting factor BOD mapping at each observation location point displayed in Table 12.

**Table 12:** Grouping of GWPR Models Based on Influential Local Factor.

Group	Influential Variables	observation location (*i*)
1	$x_{2}$	Anggana (1), East Kalimantan Province Governor's Office (9), and Pulau Kumala (15)
2	$x_{5}$	Bloro (2)
3	$x_{7}$	Palaran (13)
4	$x_{2}$, and $x_{4}$	Batoq Kelo (3), Kedang Kepala Village (4), Siran Village (6), Pampang Village Bridge (7), and Pampang Dalam (14)
5	$x_{1,}x_{2,}$ and $x_{6}$	Sebelimbingan Village (5), and Kalamur (8),
6	$x_{2,}x_{3,}$ and $x_{6}$	Muara Sungai Belayan (11)
7	$x_{2,}x_{4,}$ and $x_{6}$	Long Bangun (10), Nyan Mahulu (12), and Tering (16)

Based on the influential local factors displayed in Table 12, the GWPR model can be classified into seven groups. Group 1 represents the GWPR model of the Mahakam River water BOD at the observation points in Anggana, the East Kalimantan Province Governor’s Office, and Pulau Kumala, with the influential variable being water pH (x₂). Group 2 is the GWPR model at the observation point in Bloro, where the influential variable is ammonia concentration (x₅). Group 3 is the GWPR model at the observation point in Palaran, with the influential variable being sulfate concentration (x₇). Group 4 is the GWPR model at the observation points in Batoq Kelo, Kedang Kepala Village, Siran Village, Pampang Village Bridge, and Pampang Dalam, with the influential variables being water pH (x₂) and nitrate concentration (x₄). Group 5 is the GWPR model at the observation points in Sebelimbingan Village and Kalamur, with the influential variables being temperature (x₁), water pH (x₂), and total suspended solid (x₆). Group 6 is the GWPR model at the observation point in Muara Sungai Belayan, with the influential variables being water pH (x₂), water color degree (x₃), and total suspended solid (x₆). Group 7 is the GWPR model at the observation points in Long Bangun, Nyan Mahulu, and Tering, with the influential variables being water pH (x₂), nitrate concentration (x₄), and total suspended solid (x₆).

**Fig. 5:**
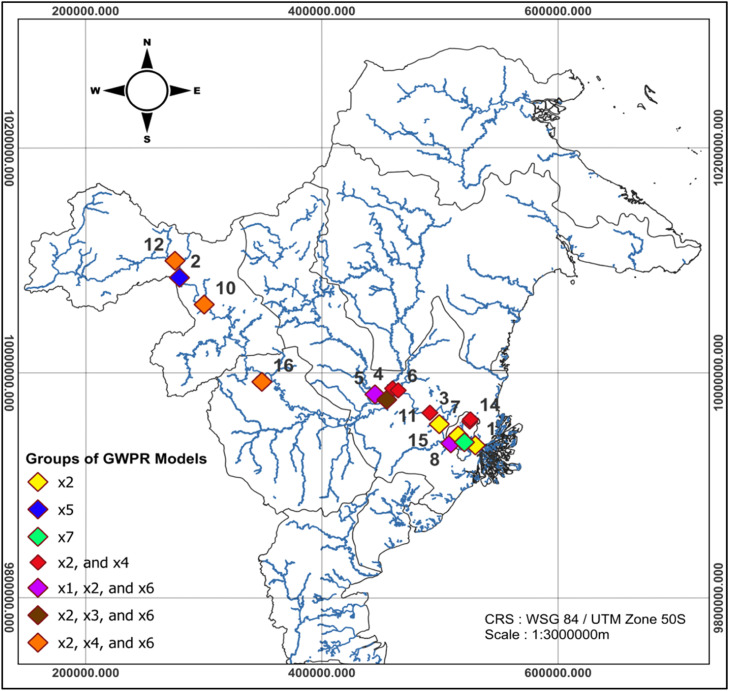
Group Distribution Map Based on the Influential Variables.

The affecting factor BOD mapping of Mahakam river water displayed in Table 12 in this research is also visualized though the distribution of the affecting factor to BOD at every observation point which can be seen in Fig. 5.

Based on Fig. 5, the GWPR parameter testing results indicate a clear spatial variation in the classification of observation points along the Mahakam River. Group 1 is represented by the observation points marked in yellow, namely the GWPR model at Anggana (*i* = 1), the East Kalimantan Province Governor’s Office (*i* = 9), and Pulau Kumala (*i* = 15), with the influential local factor being x₂. These three observation points are adjacent locations within the Samarinda City and Tenggarong areas. Group 2 is represented by the observation point marked in blue, namely the GWPR model at Bloro (*i* = 2), with the influential local factor being x₅. This observation point is located within the Kutai Kartanegara Regency area. Group 3 is represented by the observation point marked in green at Palaran (*i* = 13), with the influential local factor being x₇. This point lies within the Samarinda City area. Group 4 is represented by the observation points marked in red, namely Batoq Kelo (*i* = 3), Kedang Kepala Village (*i* = 4), Siran Village (*i* = 6), Pampang Village Bridge (*i* = 7), and Pampang Dalam (*i* = 14), with the influential local factors being x₂ and x₄. These observation points are located across two adjacent regions, namely the Kutai Kartanegara Regency area (Batoq Kelo, Kedang Kepala Village, and Siran Village) and the northern part of Samarinda City (Pampang Village Bridge and Pampang Dalam). Group 5 is represented by the observation points marked in purple, namely Sebelimbingan Village (*i* = 5) and Kalamur (*i* = 8), with the influential local factors being x₁, x₂, and x₆. These two observation points are neighboring locations in the northern part of Kutai Kartanegara Regency. Group 6 is represented by the observation point marked in brown at Muara Sungai Belayan (*i* = 11), with the influential local factors being x₂, x₃, and x₆. This observation point is located in the central part of Kutai Kartanegara Regency. Group 7 is represented by the observation points marked in orange, namely Long Bangun (*i* = 10), Nyan Mahulu (*i* = 12), and Tering (*i* = 16), with the influential local factors being x₂, x₄, and x₆. These observation points are located in the Mahakam Ulu and West Kutai Regency areas. Based on the factor mapping in Fig. 5, it can be concluded that the factors influencing Mahakam River water BOD in each district/city area are different, and generally the influencing factor of the BOD at adjacent observation points in an area of the district/city area the same.

*9. Error Homoscedasticity Testing of the GWPR Model*

The first assumption test for the GWPR model is error homoscedasticity testing. The homoscedasticity testing in *the research uses Glejser approach. The hypothesis of error homoscedasticity testing is*

$H_{0}$: $\sigma_{it}^{2}=\sigma_{2t}^{2}=\ldots =\sigma_{16t}^{2}=\sigma^{2};t=1,2,3$ (Error variance is constant across locations)
$H_{1}$: at least one $\sigma_{it}^{2}\neq \sigma^{2};i=1,2,\ldots ,\;16;t=1,2,3$ (Error variance isn’t constant across locations)

The test statistic is given by Eq. (27), and the calculation results of error homoscedasticity testing is presented in Table 13.

**Table 13:** Glejser Test Results for GWPR Model.

	$F_{\left(0,95;8;24\right)}$	$p_{5}$	*Decision*
0.8853	2.7141	0.5268	$H_{0}$ is Accepted

Based on Table 13, $H_{0}$ is accepted at the significance level of 0.05, and it can be concluded that error variance is constant across locations. The decision is confirmed by $F_{3}=0.8853>F_{\left(0,95;8;24\right)}=2.7141$, and $p_{5}=0.5268>\;\alpha =0,05$. This test confirms the GWPR model satisfies the homoscedasticity assumption of the linear regression model.

*10. The Error Normality Testing of GWPR Model*

The second assumption test for the GWPR model is the error normality testing. Normality testing in the research use Jarque-Bera (JB) approach. The hypothesis of error normality testing is

$H_{0}$: Error is normally distributed with $E(\varepsilon_{it})=0$
$H_{1}$: Error is not normally distributed

The test statistic is given by Eq. (29), and the calculation results of error normality testing is presented in Table 14.

**Table 14:** Jarque-Bera Test Results for the GWPR Model.

JB	$\chi_{\left(0.095;2\right)}^{2}$	$p_{6}$	*Decision*
2.7590	5.9915	0.2517	$H_{0}$ is Accepted

Based on Table 14, $H_{0}$ is accepted at the significance level of 0.05, it can be concluded that the error of GWPR model is normally distributed with zero mean. The decision is confirmed by the JB statictic JB=2.7590<5.9915, and $p_{6}=0,2517>\alpha =0,05$. This test confirms that the GWPR model satisfies the normality assumption of the linear regression framework.

*11. The Error Autocorrelation Testing of the GWPR Model*

The error autocorrelation of the GWPR Model in the research uses Moran’s I test. The hypothesis of the test is:

H₀ : the FEM Error are spatially independent
H₁ : the FEM Error are spatially dependent

The test statistic is given by Eq. (33), and the calculation results of homoscedasticity testing are presented in Table 15.

**Table 15:** Moran’s I Test Result for GWPR.

	*Moran’s I*	$p_{value}$	*Decision*
1.1906	−0.0436	0.2338	$H_{0}$ is Accepted

Based on Table 15, H₀ is accepted at the significance level of 0.05, and it can be concluded that the error of GWPR IS spatially independent. Its mean the error do not exhibit spatial autocorrelation. The decision is confirmed by $Moran,s\;I=-0.0436,\;|Z_{I}|=1.1906<Z_{\left(1-0.025\right)}=1.96\;$and $p_{value}=0.2338>\alpha =0.05$. Based on the results of the spatial autocorrelation test, the GWPR model errors are confirmed to be spatially independent. This indicates that the error at one observation point does not influence the errors at other observation points. Therefore, the GWPR model errors satisfy the non-autocorrelation assumption of the linear regression framework. The error analysis indicates that the GWPR model satisfies the assumptions of linear regression, thereby confirming its validity as a local linear regression model.

*12. Goodness-of-fit Measure of the FEM and GWPR Models*

The measure of model goodness in this research is the coefficient of determination (R^2^) and Root Mean Square Error (RMSE). The R^2^and RMSE value of FEM and GWPR model are displayed in Table 16.

**Table 16:** Goodness-of-fit Measures of the FEM and GWPR Model.

*Model*	*AIC*	$R^{2}$	*RMSE*
*FEM*	*5.1492*	*71.751%*	*0.9507*
*GWPR*	*−60.6419*	*80.321%*	*0.7122*

Based on the goodness-of-fit measures in Table 15, it can be concluded that the GWPR model outperforms the FEM model. It is confirmed by AIC and RMSE value of the GWPR model respectively is –60.6419 and 0.7122 which is smaller than those of the FEM model (5.1492 and 0.9507). The R² of the GWPR model is 80.321 %, which is higher than the FEM model at 71.751 %.

The results of this research show that the factors influencing BOD in the Mahakam River vary locally across observation sites. Therefore, preventive measures to increase BOD are carried out locally. Several recommendations to prevent the increasing the river water BOD and preventing river water pollution based on the results of this research are as follows. Nitrate, ammonia, and sulfate have a positive relationship with BOD. High levels of these substances are suspected to originate from domestic waste, industrial waste, and the disposal of organic materials into the river. It is recommended that communities and industries improve waste management, treat effluents before discharge, and avoid dumping directly into rivers. Local governments should strictly supervise waste management by consistently enforcing established regulations.

Total suspended solids (TSS) also show a positive relationship with BOD, caused by erosion from land use, construction, and former mining areas. Controlling sediment input can be achieved through reforestation, soil stabilization, and proper land management. Similarly, water color is positively related to BOD, largely due to erosion from former mining areas. Reforesting degraded areas and managing vacant mining lands, especially in Kutai Barat, can help reduce erosion and organic matter input.Temperature has a positive relationship with BOD because higher water temperatures accelerate microbial activity and organic matter decomposition, increasing oxygen demand. Thermal pollution can be prevented by managing industrial effluents and providing shading along riverbanks where possible. Water pH also impacts BOD, as extreme water pH values can change microbial activity. Maintaining water pH within an optimal range requires monitoring water quality and controlling waste discharge. Overall, managing BOD in the Mahakam River requires an integrated approach that addresses chemical factors (nitrate, ammonia, sulfate), physical factors (TSS, water color, temperature), and biological factors (microbial decomposition affected by pH). This approach should be combined with strict enforcement of waste management and rehabilitation of degraded areas.

*13. Comparison with External Data from Other River Observation Points*

To validate the robustness of the model and BOD patterns observed in the Mahakam River, a comparison was made using external data from other river observation points with results as follows. The parameter estimates of the local BOD model using GWR model for external data from other river observation point are presented in Table 17. Based on Table 17, the comparison using external data from other river observation points in East Kalimantan shows consistent spatial behavior of the GWPR parameters, confirming that the factors influencing BOD concentration in the Mahakam River are also evident in other river systems of tropical humid regions.

**Table 17:** Parameter Estimates from External River Data for Comparison.

Location	Test Statistics	Parameter						
		$\beta_{1}$	$\beta_{2}$	$\beta_{3}$	$\beta_{4}$	$\beta_{5}$	$\beta_{6}$	$\beta_{7}$
Sei Sepaku Brige	Estimation	0.14230	−2.07901	0.01214	−0.21198	1.92096	0.01600	−0.00849
	Standard Error	0.10418	0.33348	0.00811	0.11801	2.95264	0.00562	0.00750
	$T_{3}$	1.36588	−6.23421	1.49738	−1.79636	0.65059	2.84498	−1.13123
	$p_{8}$	0.19828	0.00005	0.16143	0.09892	0.52819	0.01542	0.28116
Nursery Suring PPU	Estimation	−0.04926	−1.89787	0.02561	−0.30698	5.59102	0.01266	−0.00734
	Standard Error	0.13788	0.37784	0.01015	0.15172	4.90777	0.00891	0.00922
	$T_{3}$	−0.35729	−5.02299	2.52163	−2.02327	1.13922	1.42005	−0.79607
	$p_{8}$	0.72739	0.00035	0.02771	0.06710	0.27795	0.18232	0.44223
Downstream (Pos Pon 1) Balikpapan	Estimation	−0.05410	−1.93252	0.02666	−0.28686	5.93151	0.01248	−0.00700
	Standard Error	0.12403	0.34222	0.00940	0.13777	4.40196	0.00812	0.00828
	$T_{3}$	−0.43613	−5.64697	2.83517	−2.08219	1.34747	1.53695	−0.84496
	$p_{8}$	0.67087	0.00013	0.01570	0.06056	0.20396	0.15155	0.41549
Upstream (Sungai 28)	Estimation	0.06404	−2.01106	0.01898	−0.26095	2.98769	0.01594	−0.00847
	Standard Error	0.11135	0.33474	0.00869	0.12554	3.47794	0.00653	0.00779
	$T_{3}$	0.57510	−6.00778	2.18548	−2.07858	0.85904	2.44262	−1.08710
	$p_{8}$	0.57638	0.00008	0.05051	0.06094	0.40799	0.03194	0.29941
Berau Regent’s Office	Estimation	−0.07607	−4.64172	0.00870	0.27690	2.72911	−0.00027	−0.05747
	Standard Error	0.19134	0.80735	0.00932	0.11886	1.99767	0.00520	0.02734
	$T_{3}$	−0.39757	−5.74930	0.93296	2.32972	1.36615	−0.05261	−2.10222
	$p_{8}$	0.69828	0.00011	0.37015	0.03910	0.19820	0.95895	0.05847
Sambaliung Berau Palace	Estimation	−0.02539	−4.65803	0.00703	0.29450	2.60454	0.00094	0.05200
	Standard Error	0.17390	0.71447	0.00847	0.11484	1.88608	0.00505	0.01775
	$T_{3}$	−0.14603	−6.51953	0.83006	2.56452	1.38093	0.18649	2.93000
	$p_{8}$	0.88645	0.00004	0.42352	0.02564	0.19374	0.85533	0.01321
Gunung Tabur Berau Palace	Estimation	−0.06772	−4.75081	0.00679	0.28990	2.60742	0.00022	0.05589
	Standard Error	0.17845	0.72863	0.00857	0.11542	1.89984	0.00511	0.01847
	$T_{3}$	−0.37946	−6.52022	0.79276	2.51167	1.37244	0.04374	3.02534
	$p_{8}$	0.71131	0.00004	0.44408	0.02821	0.19629	0.96587	0.01111
Gunung Tabur Berau Bridge	Estimation	−0.05594	−4.72585	0.00684	0.29141	2.60700	0.00043	0.05481
	Standard Error	0.17707	0.72410	0.00854	0.11524	1.89494	0.00509	0.01824
	$T_{3}$	−0.31594	−6.52651	0.80112	2.52867	1.37577	0.08423	3.00474
	$p_{8}$	0.75775	0.00004	0.43942	0.02736	0.19529	0.93433	0.01153
Downstream Berau Coal	Estimation	−0.07301	−4.70301	0.00778	0.28293	2.64986	0.00007	0.05608
	Standard Error	0.18445	0.76438	0.00889	0.11699	1.93690	0.00514	0.02066
	$T_{3}$	−0.39582	−6.15270	0.87463	2.41837	1.36809	0.01276	2.71504
	$p_{8}$	0.69954	0.00006	0.39980	0.03336	0.19761	0.99004	0.01952
Upstream PT. BBE Berau	Estimation	−0.04881	−4.70963	0.00689	0.29211	2.60621	0.00055	0.05416
	Standard Error	0.17644	0.72240	0.00854	0.11528	1.89282	0.00509	0.01814
	$T_{3}$	−0.27662	−6.51941	0.80719	2.53403	1.37690	0.10760	2.98594
	$p_{8}$	0.78702	0.00004	0.43606	0.02709	0.19495	0.91618	0.01193

At the Sei Sepaku Bridge, Nursery Suring PPU, Downstream Balikpapan, and Upstream Sungai 28 observation points, water pH (x₂) consistently appears as a significant factor (|T₃| > 5.0; p₈ < 0.001), indicating a strong negative effect on BOD concentration. This pattern shows that higher pH values are generally associated with lower BOD levels. In addition, color degree (x₃) and TSS (x₆) also show significant positive effects at several of these locations, suggesting that increased organic coloration and suspended solids correspond to higher organic pollution loads. These results demonstrate that both chemical (pH) and physical (color and TSS) parameters jointly influence organic pollution in the downstream and midstream zones of East Kalimantan’s rivers.

In the Berau region covering the points at Berau Regret’s Office, Sambaliung Palace, Gunung Tabur Palace, Gunung Tabur Bridge, Downstream Berau Coal, and Upstream PT. BBE the results show a very consistent pattern. Across all these sites, water pH (x₂) strongly and negatively affects BOD (|T₃| ≈ 6.1–6.5; p₈ < 0.001). Moreover, nitrate concentration (x₄) and sulfate concentration (x₇) also emerge as significant predictors (p₈ < 0.05) in most locations, particularly at Sambaliung Palace, Gunung Tabur Palace, and Downstream Berau Coal. The positive direction of these coefficients indicates that increased nitrate and sulfate levels correspond to higher BOD concentrations, reflecting the impact of nutrient enrichment and industrial effluents.

Overall, water pH (x₂) and nitrate concentration (x₄) consistently show significant effects across multiple observation points, while sulfate concentration (x₇) plays an additional role in the Berau sub-region. These findings confirm that the main controlling factors of BOD in East Kalimantan’s tropical humid rivers are chemical and hydrological parameters. The consistent spatial pattern and cross-location validation indicate that the Mahakam River GWPR model is robust and spatially adaptive, as it can capture both general trends and local variations that naturally occur across different watersheds.

Based on the geographically weighted panel regression (GWPR) modeling of BOD data, this study concludes that the GWPR model outperforms the global fixed-effects model (FEM). The GWPR model effectively captures and maps the local factors influencing BOD in the Mahakam River at each observation site. These locally varying factors include water temperature, pH, color degree, nitrate concentration, ammonia concentration, total suspended solids (TSS), and sulfate concentration.

## Limitations

‘Not applicable’

## Ethics statements

The research data is secondary data, namely the Analyze Report of the Surface Water Data Quality Monitoring from the Life Environmental Department of the East Kalimantan Province 2022–2024. The research sample size was 16 observation points with 48 observation data within 3 periods in 2022–2024. The research data consists of the response variable data, namely BOD (y), and predictor variable data, namely temperature $\left(x_{1}\right)$, water pH$\;(x_{2})$, water color degree $\left(x_{3}\right)$, nitrate concentration $\left(x_{4}\right)$, ammonia concentration $\left(x_{5}\right)$, total suspended solid or TSS $\left(x_{6}\right)$, sulfate concentration $\left(x_{7}\right)$, also coordinate data of observation location points. Data processing and analysis were performed using RStudio 4.3.1, GNU Octave 9.3.0, and QGIS 3.32.3.

## Declaration of competing interest

The authors declare that they have no known competing financial interests or personal relationships that could have appeared to influence the work reported in this paper.

## Data Availability

No data was used for the research described in the article.
